# Acute Ketamine Facilitates Fear Memory Extinction in a Rat Model of PTSD Along With Restoring Glutamatergic Alterations and Dendritic Atrophy in the Prefrontal Cortex

**DOI:** 10.3389/fphar.2022.759626

**Published:** 2022-03-17

**Authors:** Nathalie Sala, Caterina Paoli, Tiziana Bonifacino, Jessica Mingardi, Emanuele Schiavon, Luca La Via, Marco Milanese, Paolo Tornese, Ashok K. Datusalia, Jessica Rosa, Roberta Facchinetti, Giulia Frumento, Giulia Carini, Floramarida Salerno Scarzella, Caterina Scuderi, Lia Forti, Alessandro Barbon, Giambattista Bonanno, Maurizio Popoli, Laura Musazzi

**Affiliations:** ^1^ Laboratory of Neuropsychopharmacology and Functional Neurogenomics, Dipartimento di Scienze Farmaceutiche, Università Degli Studi di Milano, Milano, Italy; ^2^ School of Medicine and Surgery, University of Milano-Bicocca, Monza, Italy; ^3^ Department of Pharmacy, Unit of Pharmacology and Toxicology, University of Genoa, Genoa, Italy; ^4^ Department of Molecular and Translational Medicine, University of Brescia, Brescia, Italy; ^5^ Department of Biotechnology and Life Sciences, University of Insubria, Busto Arsizio, Italy; ^6^ Department of Pharmacology and Toxicology, National Institute of Pharmaceutical Education and Research (NIPER), Raebareli, India; ^7^ Department of Pharmacology, Medical School of Ribeirão Preto, University of São Paulo, Ribeirao Preto, Brazil; ^8^ Department of Physiology and Pharmacology “Vittorio Erspamer”, SAPIENZA University of Rome, Rome, Italy; ^9^ IRCCS Ospedale Policlinico San Martino, Genoa, Italy

**Keywords:** acute stress, ketamine, PTSD, prefrontal cortex (PFC), glutamate transmission, dendritic arborization, fear extinction, animal model

## Abstract

Stress represents a major risk factor for psychiatric disorders, including post-traumatic stress disorder (PTSD). Recently, we dissected the destabilizing effects of acute stress on the excitatory glutamate system in the prefrontal cortex (PFC). Here, we assessed the effects of single subanesthetic administration of ketamine (10 mg/kg) on glutamate transmission and dendritic arborization in the PFC of footshock (FS)-stressed rats, along with changes in depressive, anxious, and fear extinction behaviors. We found that ketamine, while inducing a mild increase of glutamate release in the PFC of naïve rats, blocked the acute stress-induced enhancement of glutamate release when administered 24 or 72 h before or 6 h after FS. Accordingly, the treatment with ketamine 6 h after FS also reduced the stress-dependent increase of spontaneous excitatory postsynaptic current (sEPSC) amplitude in prelimbic (PL)-PFC. At the same time, ketamine injection 6 h after FS was found to rescue apical dendritic retraction of pyramidal neurons induced by acute stress in PL-PFC and facilitated contextual fear extinction. These results show rapid effects of ketamine in animals subjected to acute FS, in line with previous studies suggesting a therapeutic action of the drug in PTSD models. Our data are consistent with a mechanism of ketamine involving re-establishment of synaptic homeostasis, through restoration of glutamate release, and structural remodeling of dendrites.

## Introduction

Post-traumatic stress disorder (PTSD) is a debilitating, often chronic and comorbid, mental illness with yet limited options for pharmacological treatment, mainly based on selective serotonin reuptake inhibitors (SSRIs). SSRIs require weeks to months to induce a clinical effect and obtain full remission in fewer than half of the treated patients (Akiki and Abdallah, 2019). Consequently, drugs with higher effectiveness and faster onset of action are urgently needed. While it is known that stress is a primary risk factor for neuropsychiatric disorders, in PTSD, the relationship with the exposure to strong, traumatic events is particularly evident (Fulton et al., 2015; Koenen et al., 2017). PTSD has been also conceptualized as a disorder involving dysfunctional processing of fear and fear memory. Fear is a primary emotion with obvious evolutionary value, which defends one’s biological integrity from actual or envisaged danger and may predispose to a fight-or-flight reaction (Popoli et al., 2012; LeDoux, 2014; McEwen et al., 2015). Fear memory has been extensively studied in rodent models, particularly by using fear conditioning protocols, which have been proposed as animal models for stress-related disorders, including PTSD (Milad and Quirk, 2012; Izquierdo et al., 2016).

In the last several years, a major shift in the conceptual framework of pathophysiology and treatment of neuropsychiatric disorders has occurred, moving from the monoamine-oriented to the neuroplasticity hypothesis, in which the role of the glutamate system is conceived as a primary mediator of psychopathology and a straight target for antidepressant drugs (Duman and Aghajanian, 2012; Sanacora et al., 2012; Musazzi et al., 2013; Abdallah et al., 2015; Murrough, 2016; Lener et al., 2017). Many preclinical and clinical studies suggest that dysregulation of glutamatergic synaptic function may be an important feature of the pathophysiology of PTSD (Krystal et al., 2018). Indeed, magnetic resonance imaging (MRI) studies in PTSD patients have revealed reduced cortical thickness and impaired cortical functional connectivity associated with PTSD symptoms and cognitive deficits, suggesting the clinical relevance of these morpho-functional alterations (O’Doherty et al., 2015). In parallel, chronic stress application in rodents has consistently shown to reduce apical dendrite length and branching of the medial prefrontal cortex (PFC) pyramidal neurons (layers II/III and V) and hippocampus CA3 pyramidal neurons (Pittenger and Duman, 2008; Leuner and Shors, 2013). Often, the dendritic atrophy in the PFC and hippocampus goes along with a reduction of the density of synaptic spines, suggesting that stress induces a “synaptic disconnection” syndrome within and between these areas. A widely shared hypothesis suggests that abnormal enhancement of glutamate release/transmission induced by stress has a primary role in the neuroarchitecture changes observed (Duman and Aghajanian, 2012; Sanacora et al., 2012; Abdallah et al., 2015; McEwen et al., 2015; Musazzi et al., 2017a; Duman et al., 2019; Tornese et al., 2019). Consistent with the hypothesis, blockade of N-methyl-D-aspartate (NMDA) receptors during repeated restraint stress prevented stress-induced apical dendritic retraction in the mPFC (Martin and Wellman, 2011).

In previous studies, we have thoroughly characterized the functional and morphological changes induced in the PFC of rats by acute inescapable footshock (FS), a widely used animal model of PTSD (Richter-Levin et al., 2019). We demonstrated that a single session of FS induces enhancement of depolarization-evoked glutamate (not GABA) release in PFC, which was rapid (observed immediately after stress exposure) and sustained for at least 24 h (Musazzi et al., 2010, 2017b; Treccani et al., 2014). FS also induced time-dependent modulation of both α-amino-3-hydroxy-5-methyl-4-isoxazolepropionic acid (AMPA) and NMDA receptor subunit expression and phosphorylation, suggesting an early and transient enhancement of AMPA receptor (AMPAR)-mediated currents, followed by a transient activation of NMDA receptors (NMDAR) (Bonini et al., 2016). Chronic antidepressants with different primary mechanisms have the common effect of preventing the overflow of presynaptic glutamate induced by acute FS, introducing a new component in the mechanism of antidepressants (Musazzi et al., 2010; Tardito et al., 2010). Interestingly, these functional alterations of glutamatergic transmission were accompanied by dendritic atrophy and retraction, observed as soon as 24 h after FS and sustained for at least 14 days (Nava et al., 2017). The pretreatment of the animals for 2 weeks with the antidepressant desipramine prevented neuroarchitecture alterations. Overall, the evidence collected clearly demonstrated that acute inescapable stress may induce rapid and long-term changes in synaptic function and neuronal architecture in PFC (Musazzi et al., 2018).

In recent years, several clinical studies, mostly with treatment-resistant depressed subjects, have shown that a single infusion of a subanesthetic dose of ketamine (NMDAR antagonist) exerts a rapid (within hours) and sustained (at least a week or longer) antidepressant effect (Zanos and Gould 2018; Gould et al., 2019; Kadriu et al., 2019). While it has been argued that the antidepressant action of subanesthetic ketamine is linked to its antagonism of NMDAR in GABAergic interneurons of PFC, which in turn stimulates excitatory (AMPAR-mediated) transmission, it has also been shown that NMDAR-independent mechanisms mediate ketamine action (Wray et al., 2019).

In 2019, intranasal administration of the S-ketamine enantiomer (esketamine) in conjunction with an oral antidepressant has been granted approval by the US FDA for the management of treatment-resistant depression. The rapid and long-lasting antidepressant action of ketamine has also raised interest as a new putative treatment for PTSD (McGowan et al., 2017; Duman et al., 2019; Liriano et al., 2019).

In the present study, we aimed at analyzing whether acute subanesthetic ketamine (10 mg/kg) can rescue the maladaptive changes induced by acute inescapable FS stress. We administered ketamine at various times before or 6 h after FS and found that both prior (at least 24 h before) and subsequent administration of ketamine completely blocked the FS-induced enhancement of glutamate release in PFC of FS-stressed rats. Moreover, ketamine given 6 h after FS also completely rescued the increase of peak amplitude in spontaneous excitatory postsynaptic current (sEPSC) in prelimbic (PL) PFC (layers II–III) pyramidal neurons from FS-stressed rats, measured 24 h after FS, and fully restored apical dendrite atrophy in PL-PFC (layers II–III) measured 1 week after FS. On the behavioral level, we found that ketamine did not change depression- (anhedonic) or anxiety-like behavior, but facilitated the extinction of contextual fear memory, a mechanism that is impaired in PTSD (Milad and Quirk, 2012; Izquierdo et al., 2016). The present results may suggest ketamine as a putative treatment to prevent the development of PTSD and other stress-related disorders.

## Materials and Methods

### Animals

All experimental procedures involving animals were performed in accordance with the European Community Council Directive 2010/63/UE and were approved by the Italian legislation on animal experimentation (Decreto Legislativo 26/2014, animal experimentation licenses 521/2015-PR, 140/2014-B—DGSAF24898, 505/2017-PR).

Adult Sprague–Dawley male rats were used (350–450 g, Charles River, Calco, Italy). For the measurement of sucrose intake, since the employed protocol requires 5 weeks (see below), at the beginning of this experiment, the animals used were 175–200 g in weight (350–450 g at the end of the protocol).

All the animals were housed two per cage and maintained on a 12/12-h light/dark schedule (lights on at 7:00 a.m.), in a temperature- and humidity-controlled facility with free access to food and water. All the animals were sacrificed by beheading.

The number of animals used in each experiment and all statistical details (including exact *p* values for all the comparisons) are reported in Supplementary Tables S1–6.

### Drug Treatments

Racemic ketamine (MSD Animal Health, Milan, Italy) 10 mg/kg, i.p., and desipramine (Merck, Milan, Italy) 10 mg/kg, i.p., both in saline 0.9%, were used.

### Footshock Stress Procedure

Animals were subjected to a single session of acute inescapable FS stress as previously reported (Musazzi et al., 2019): intermittent shocks (0.8 mA) for 40 min (20 min total of actual shock with random intershock length between 2 and 8 s). The FS box was connected to a scrambler controller (LE 100-26, Panlab) that delivers intermittent shocks to the metallic floor. Control animals were left undisturbed in their home cages.

### Preparation of Purified Synaptosomes and Neurotransmitter Release Experiments

Three experimental protocols were performed to study the effect of ketamine on endogenous glutamate and GABA release.

In the first set of experiments, we assessed the effect of ketamine on glutamate and GABA neurotransmission. Rats were randomly assigned to i.p. injection with saline (0.9% NaCl, control), desipramine (DMI—conventional antidepressant used as positive control), or ketamine and sacrificed 1, 2, 6, 24, 72 h or 1 week after. Five–15 animals/group were used.

In the second set of experiments, we assessed the preventive effect of ketamine treatment on FS-induced neurotransmitter release alteration. Rats were randomly divided in four experimental groups: 1) animals injected with saline and left undisturbed in their home cages (control), 2) animals injected with saline and subjected to FS, 3) animals injected with ketamine and left undisturbed in their home cages, and 4) animals injected with ketamine and subjected to FS. Rats were injected (saline or ketamine) 2, 24, or 72 h before the stress session and sacrificed immediately after the 40 min of FS procedure. Five–15 animals/group were used.

In the third set of experiments, we assessed the restoring effect of ketamine treatment after FS on neurotransmitter release alteration. Rats were randomly assigned to three groups: 1) animals injected with saline and not subjected to FS, 2) animals injected with saline and subjected to FS, and 3) animals injected with ketamine and subjected to FS. Rats were injected (saline or ketamine) 6 h after FS start and were sacrificed 24 h after FS start. Ten animals/group were used.

Synaptic terminals (synaptosomes) were freshly prepared from homogenized PFC (whole frontal lobe) by centrifugation on discontinuous Percoll gradients as previously described (Stigliani et al., 2006). Aliquots of the synaptosomal suspensions were distributed on microporous filters placed at the bottom of a set of parallel superfusion chambers maintained at 37°C and superfused with a standard physiological medium (140 mM NaCl, 3 mM KCl, 1.2 mM MgSO_4_, 1.2 mM CaCl_2_, 1.2 mM NaH_2_PO_4_, 5 mM NaHCO_3_, 10 mM glucose, and 10 mM HEPES, pH 7.4) (Raiteri et al., 1984; Bonanno et al., 2005). After 36 min of superfusion, samples were collected as follows: two 3-min samples (*t* = 36–39 and 45–48 min; basal release) before and after and one 6-min sample (t = 39–45 min; stimulus-evoked release). Stimulation with a 90-s pulse of 15 mM KCl was applied at t = 39 min. Quantification of endogenous glutamate GABA was determined by high-performance liquid chromatography (HPLC) as previously described (Raiteri et al., 2000). The stimulus-evoked overflow was estimated by subtracting glutamate or GABA content of the two 3-min basal release samples from the content of the 6-min sample collected during and after the stimulating pulse.

### Electrophysiology

For patch-clamp whole-cell recordings, coronal brain slices (300 μm; 9–13-week-old rats) containing the PL-PFC were maintained in an interface chamber with carboxygenated saline containing the following (in millimolar): NaCl (83), KCl (2.5), NaH_2_PO_4_ (1.25), NaHCO_3_ (21), glucose (25), sucrose (72), Na-ascorbic acid (0.45), CaCl_2_ (1), and MgCl_2_ (4). During recordings, slices were perfused (2 ml/min) with carboxygenated artificial cerebrospinal fluid (aCSF; 33 ± 1°C) containing the following (in millimolar): NaCl (125), KCl (2.5), NaH_2_PO_4_ (1.25), NaHCO_3_ (26), D-glucose (10), CaCl_2_ (1.5), and MgCl_2_ (1). Data were acquired with a Multiclamp 700B, low-pass filtered at 10 KHz and sampled at 50 KHz using Digidata 1440A and pClamp10 (Molecular Devices). For miniature excitatory postsynaptic current (mEPSC) recordings (at −60 mV), whole-cell pipettes contained the following (in millimolar): K-gluconate (132), EGTA (0.2), HEPES (10), Na_2_-ATP (4), Na_3_GTP (0.3), Na_2_-phosphocreatine (5), MgCl_2_ (4), and KCl (8), pH 7.4 (“KGluc” solution). Slices were continuously perfused with tetrodotoxin (TTX, 1 µM), picrotoxin (0.1 mM), and strychnine (1 μM). Nine–14 animals/group were used.

For spontaneous excitatory or inhibitory postsynaptic current (sEPSC/sIPSC) recordings, whole-cell pipettes contained the following (in millimolar): CsMeSO_3_ (132), EGTA (0.2), HEPES (10), Mg-ATP (4), Na_3_GTP (0.3), Na_2_-phosphocreatine (5), MgCl_2_ (4), KCl (10), and lidocaine N-ethyl chloride (5), pH 7.4 (“Cs-based” solution). sEPSCs and sIPSCs were isolated in individual cells by recording at the reversal potential of sIPSCs (−58 mV) and sEPSCs (+3 mV), respectively (Maffei et al., 2004). No correction was applied for liquid junction potentials. Neurons with compensated access resistance (*R*_a_) >14 MΩ or *R*_a_ changes >30%, resting potential >−60 mV, or input resistance (*R*_in_) <50 MΩ were not analyzed. Putative pyramidal cells were identified from their large, triangular-shaped cell body in layers II–III of PL-PFC, 70–130 µm from the pial surface (van Aerde and Feldmeyer, 2015). To further exclude potential interneurons, cells with *R*_in_ ≥200 MΩ (with KGluc solution) (Wang and Gao, 2009) or ≥250 MΩ (with Cs-based solution) were not analyzed. In the KGluc recordings, neuronal firing was also assessed to check for fast-spiking interneurons (Wang and Gao, 2009). Postsynaptic currents (PSCs) were detected in 120-s-long intervals (∼15, ∼5, or ∼8 min after break-in for mEPSCs, sEPSCs, or sIPSC, respectively) with Clampfit 10 (MDS Analytical Technologies), using the template search routine (match threshold, mEPSCs: 4, sEPSCs: 2.5, sIPSCs: 3.5; peak threshold: 10 pA for mEPSCs and sIPSCs; 12 pA for sEPSCs). Five–nine animals/group were used.

### Golgi-Cox Staining and Dendritic Analysis

A total of 18 rats were included in this study, 12 of which were randomly selected to undergo FS stress. Six hours after the start of the stress protocol, half of the stressed animals were injected with ketamine while the other animals with saline. All the rats were sacrificed 7 days after the stress session, and immediately after sacrifice, left or right hemispheres were randomly processed for Golgi-Cox staining using the Rapid Golgi Stain Kit (FD NeuroTechnologies, Inc., Columbia, MD, United States) (Tornese et al., 2019). Hemispheres were coronally sliced (200 μm) on a cryostat (Leica CM1950, Leica Biosystems, Buccinasco, Italy).

The day after cutting, sections were stained, dehydrated through a graded series of ethanol, cleared in xylene, and covered with Eukitt^®^ coverslip (Carlo Erba, Cornaredo, Italy). Layers II–III within PL-PFC was identified as described previously (Nava et al., 2017) using a ×20 objective on a light microscope (Nikon ViCo, Nikon Instruments S.p.A., Firenze, Italy). Using a ×40 objective, pyramidal neurons were identified by dendrites extending into two distinct conical arbors (biconical radiation). Z-stacks (80–100 μm; Z-step size 1 μm) of three–six pyramidal neurons/animal with untruncated branches were acquired (Nikon ViCo, Nikon Instruments S.p.A., Firenze, Italy).

Images were converted to 8-bit and processed using the “Image Stitching” plugin of the open-source Fiji software (Preibisch et al., 2009). Images were then binarized and paths traced manually using the “Simple Neurite Tracer” Fiji plugin (Longair et al., 2011). The total length of the apical dendritic arbor was expressed in micrometers. Three–five neurons/animal were analyzed. The same images were processed for the analysis of apical dendritic spine density. An 80–100-µm segment was traced on an apical secondary dendrite using the “Simple Neurite Tracer” Fiji plugin, and spines were counted manually. The number of spines/10 µm was calculated for each neuron. All the images were processed by two operators blind to the experimental groups.

### Primary Neuronal Cultures: Transfection, Treatments, and Confocal Imaging Analysis

Primary cultures of cortical neurons were prepared as previously described (Bonini et al., 2015). Cortices from day 16.5 embryos were dissociated mechanically, and neurons were resuspended in Neurobasal™ medium supplemented with B27 (Gibco™, Thermo Fisher Scientific, Rodano, Italy) containing 30 U/ml penicillin, 30 mg/ml streptomycin (Merck), 0.75 mM GlutaMAX (Gibco™, Thermo Fisher Scientific), and 0.75 mM L-glutamine (Gibco™, Thermo Fisher Scientific) (animal experimentation license N 480/2017-PR). Neurons were seeded in two-well Lab-Tek^®^ II (Nunc^®^, Thermo Fisher Scientific) coated with 0.1 mg/ml Poly-D-lysine at a density of 210.500 cells/4 cm^2^ and maintained at 37°C under a 5% CO_2_ humid atmosphere. Three days after seeding, half of the medium was replaced with an astrocyte-conditioned medium. Afterward, half of the medium was changed every 7 days up to a maximum of 4 weeks.

Neurons were transfected with an enhanced green fluorescent protein (EGFP)-expressing vector using Magnetofection™ Technology at day *in vitro* (DIV)11 (Oz Biosciences, Marseille, France), according to the manufacturer protocol. Images of fluorescently labeled living pyramidal neurons were acquired at DIV17 (T0) using an LSM880 inverted confocal microscope (Zeiss, Jena, Germany) with a 20X objective in a humid chamber at 37°C and 5% CO_2_. Cells were then incubated with 500 nM corticosterone (Merck) in 0.2% dimethyl sulfoxide (DMSO) (Sigma-Aldrich) or 0.2% DMSO, for 20 min. Immediately after, corticosterone-treated cells were exposed to a complete wash-out with an astrocyte-conditioned medium or 1 µM ketamine in an astrocyte-conditioned medium, for 1 h. Cells incubated with DMSO were also washed-out with an astrocyte-conditioned medium for 1 h (control). All the cells were then washed with 1X HBSS and fixed in 4% PFA before being used for a second session of image acquisition (T1).

Images analyzed represent maximum intensity projections of eight consecutive optical sections taken at a 0.96-μm interval with a resolution of 3,964 (x/y) pixel. Total dendritic lengths of each cell at T0 and T1 were traced using the Simple Neurite Tracer Fiji Plugin (Longair et al., 2011) in a minimum of 10 cells for each condition in two–three independent experiments, with the operator blind to treatment paradigm. The difference between T1 and T0 measurements within the same cell was calculated and expressed as Δ dendritic length T1–T0 (micrometers).

### Sucrose Intake Test

The sucrose intake test was performed essentially as in Christensen et al. (2011) with little changes. Rats were habituated to a palatable sweet solution by removing water and exposing them to two bottles with 1% sucrose solution for 2 h. Starting from the day after habituation, animals were exposed to sucrose twice a week for 4 weeks, according to the following protocol: rats were single housed, provided with two bottles, one containing 1% sucrose and one containing tap water (the position of the bottles was inverted after 30 min), with no food pellet, for 1 h. Animals were not food and/or water deprived before the test. The average sucrose solution volume drunk by each animal was calculated and defined as baseline sucrose intake. After 4 weeks, animals were randomly assigned to FS or left undisturbed in their home cages (control). 6 h after the stress session, half of the FS-stressed animals were injected with ketamine, while the others with saline. Sucrose intake (volume of sucrose solution drunk) was measured 24, 48, 72 h and 1 week after FS exposure, testing the rats as before (single housing, two bottles, one containing 1% sucrose and one containing tap water, with no food pellet, for 1 h). Data were expressed as percent sucrose intake vs. baseline sucrose intake for each animal. Eight–10 animals/group were used.

### Novelty-Suppressed Feeding

The same animals used in the sucrose intake test underwent the novelty-suppressed feeding (NSF) test 8 days after the FS stress session (24 h after the last sucrose intake test).

NSF was performed essentially as previously described (Ieraci et al., 2016). Briefly, rats were food deprived for 24 h and individually put in a corner of a square arena (60 × 60 × 40 cm), where a regular chow pellet was placed in the center on a white paper platform, under full bright light. The latency to the first bite was recorded. Eight–10 animals/group were used.

### Fear Conditioning and Extinction

Animals were exposed to FS (fear conditioning) or sham stress (rats were placed in the FS apparatus, without receiving the shock), and half of the stressed animals were injected with ketamine (the others with saline) 6 h after the start of stress. Fear extinction was performed, placing rats in the same cage in which they received FS for 5 min, every day, for several subsequent days. Percent freezing time, defined as absence of movement except for respiration, was measured by an operator blind to the experimental paradigm.

Two experiments have been performed: in the first experiment, extinction sessions were performed 1, 2, 3, and 4 days after stress, while in the second experiment, extinction sessions were performed 3, 4, 5, 6, and 7 days after stress. Twelve–14 animals/group were used in the first experiment, while 10–13 animals/group were used in the second experiment.

### Statistics

All the data were tested for normality using the Kolmogorov–Smirnov normality test with Dallal–Wilkinson–Lilliefor *p* value.

Time-course experiments of glutamate and GABA release after acute desipramine and ketamine treatment and release experiments in animals subjected to FS after ketamine treatment were compared through two-way analysis of variance (ANOVA) followed by Bonferroni *post*-*hoc* test. One-way ANOVA followed by the Bonferroni *post*-*hoc* test was applied to all the other experiments except for electrophysiological measurements, sucrose intake, and fear extinction.

Normalized binned cumulative histograms of EPSC peaks and inter-event intervals were compared across CNT, FS, and FS-KET cells using two-way repeated measures (RM)-ANOVA, followed by Tukey’s multiple comparisons test. The means of peaks and inter-event intervals were compared across CNT, FS, and FS-KET groups with one-way ANOVA or the non-parametric Kruskal–Wallis test, depending on results of normality tests, followed by multiple comparisons tests as specified.

Mixed-effects model was used for sucrose intake test, followed by Tukey’s multiple comparisons test. Fear extinction experiments were analyzed through two-way RM-ANOVA, followed by Tukey’s multiple comparisons test.

Data were expressed as mean ± standard error of the mean (S.E.M.). Statistical analysis was carried out using GraphPad Prism9 (GraphPad Software, La Jolla, CA, United States).

## Results

### Subanesthetic Ketamine Blocks the Enhancement of Glutamate Release in the Prefrontal Cortex of Rats Subjected to Acute Footshock Stress

**FIGURE 1:**
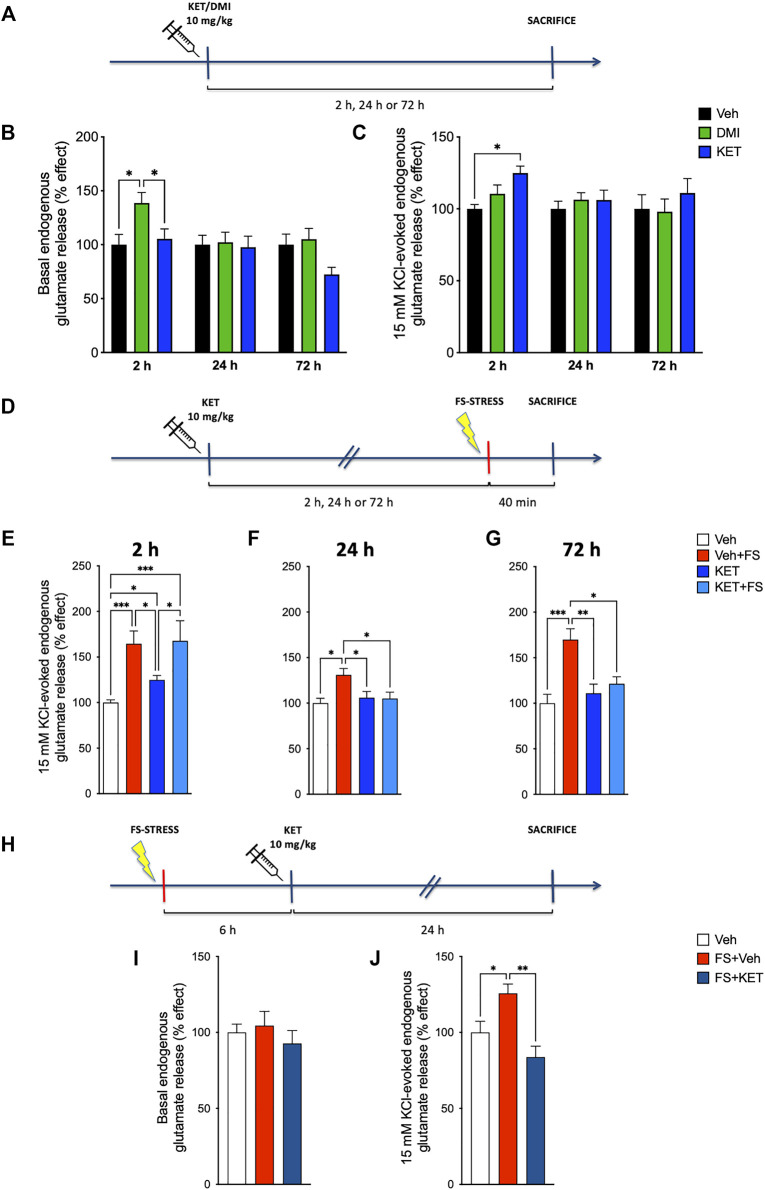
Endogenous glutamate release: **(A)** experimental plan timeline for panels **(B)** and **(C)**. Rats were injected with saline (vehicle, Veh), ketamine (KET, 10 mg/kg), or desipramine (DMI, 10 mg/kg) and sacrificed 2, 24, or 72 h after. Time-course of changes in basal **(B)** and 15 mM KCl-evoked **(C)** endogenous glutamate release from prefrontal cortex (PFC)-purified synaptosomes in superfusion. The net depolarization-evoked overflow was calculated by subtracting the transmitter content of the basal outflow. Data are expressed as percent change means ± standard error of the mean (S.E.M.) vs. Veh. Two-way analysis of variance (ANOVA), Bonferroni *post-hoc* test. *p < 0.05. **(D)** Experimental plan timeline for panels **(E–G)**. Rats were injected with saline (Veh), injected with saline and subjected to acute footshock (Veh + FS), injected with ketamine (KET), or injected with ketamine and subjected to acute FS (KET + FS). FS was administered 2, 24, or 72 h after Veh/KET injection, and animals were sacrificed immediately after the FS session. Changes in 15-mM KCl-evoked endogenous glutamate release from PFC-purified synaptosomes in superfusion of FS-stressed rats measured 2 h **(E)**, 24 h **(F)**, or 72 h **(G)** after Veh/KET injection. Data are expressed as percent change means ± SEM vs. Veh. One-way ANOVA, Bonferroni *post-hoc* test. *p < 0.05, **p < 0.01, ***p < 0.01, and ****p < 0.001. **(H)** Experimental plan timeline for panels **(I,J)**. Rats were injected with saline (Veh), subjected to acute FS, and injected with saline 6 h after stress (FS + Veh) or subjected to acute FS and injected with ketamine 6 h after stress (FS + KET). All the animals were sacrificed 24 h after FS beginning. Changes in basal **(I)** and 15-mM KCl-evoked **(J)** endogenous glutamate release from PFC-purified synaptosomes in superfusion. Data are expressed as percent change means ± SEM vs. Veh. One-way ANOVA, Bonferroni *post-hoc* test. *p < 0.05 and **p < 0.01.

First, as the enhancement of glutamate release is a key change in the induction of maladaptive changes in neuroarchitecture (see above), we sought to investigate the effect of ketamine on glutamate release in naïve rats. A pioneer study of Moghaddam et al. (1997) showed that acute administration of sub-anesthetic ketamine in naïve rats induces rapid and transient increase of extracellular glutamate in PFC, as measured by *in vivo* microdialysis. Although this effect on glutamate has been suggested as a main component in the antidepressant action of ketamine (Kavalali and Monteggia, 2012; Lener et al., 2017; Duman et al., 2019; Kadriu et al., 2019), it has not been replicated with other, more specific methods. To this end, we used the technique of purified synaptosomes in superfusion, a method that allows accurate characterization of the presynaptic release of endogenous amino acid neurotransmitters (Raiteri et al., 1984; Raiteri et al., 2000; Bonanno et al., 2005), to compare the time-dependent effects of acute sub-anesthetic ketamine (10 mg/kg, i.p.) with those of acute desipramine (10 mg/kg, i.p.), a reference antidepressant, or vehicle (saline solution, control) on spontaneous (basal) and depolarization-dependent presynaptic glutamate release in the PFC of rats (Figures 1A–C). With regard to basal glutamate release, two-way ANOVA revealed a significant effect of time [*F*(2,95) = 3.975, *p* < 0.05] and treatment [*F*(2,95) = 3.798, *p* < 0.05] (Figure 1B). Compared to vehicle-treated rats, basal glutamate release was significantly higher 2 h after desipramine injection; ketamine did not induce changes of basal glutamate release at the observed time points (Bonferroni *post-hoc* test, BPHT). Instead, we found a slight increase of depolarization-evoked glutamate release 2 h after ketamine injection and no significant effects of desipramine (Figure 1C) [significant effect of treatment *F*(2,101) = 3.045, *p* < 0.05; BPHT]. GABA basal and depolarization-evoked release was unchanged (Supplementary Figures S1A–C). To preliminary test the effects of ketamine and desipramine on glutamate release at other time points, we performed pilot experiments injecting rats with the two drugs and measuring basal and depolarization-evoked glutamate release from superfused PFC synaptosomes 1 h, 6 h, and 1 week after (*N* = 3–5, Supplementary Figure S2). We observed an increase of basal glutamate release 1 h after ketamine (Kruskal–Wallis test, 5.964, *p* < 0.05; Dunn’s multiple comparison test) and 6 h after desipramine injection (Kruskal–Wallis test, 4.600, *p* < 0.05; Dunn’s multiple comparison test).

Second, we investigated the effect of ketamine in FS-stressed rats. In previous studies, we consistently reported that acute inescapable FS stress induces a rapid and long-lasting increase of depolarization-evoked glutamate release in PFC (Musazzi et al., 2017b; Musazzi et al., 2017a) and that the chronic pretreatment with traditional antidepressants prevents the enhancement of glutamate release (Musazzi et al., 2013). Here, we asked whether the acute pretreatment with ketamine may exert similar effects on the increase of depolarization-evoked glutamate release measured in PFC synaptosomes immediately after the 40 min of FS. When given 2 h before FS (Figure 1E), ketamine did not reduce the enhancement of glutamate release induced by acute stress. Instead, if injected 24 or 72 h before stress exposure (Figures 1F,G, respectively), ketamine abolished the stress-dependent increase of glutamate release evoked by depolarization [two-way ANOVA; 2 h: significant effect of stress *F*(1,38) = 28.45, *p* < 0.001; 24 h: significant effect of stress × treatment interaction *F*(1,38) = 4.508, *p* < 0.05; 72 h: significant effect of stress *F*(1,39) = 10.80, *p* < 0.01; and stress × treatment interaction *F*(1,39) = 5.919, *p* < 0.05; BPHT]. It is worth mentioning that ketamine in naïve rats increased glutamate release at 2 h, as observed already in Figure 1C. Basal and depolarization-evoked GABA releases were not affected by either FS or ketamine treatment (Supplementary Figures S1D–G).

Third, considering the previous evidence showing that the increase of glutamate release induced by FS lasts at least 24 h after the stress session (Musazzi et al., 2017b), we assessed whether acute ketamine was able to abolish excess glutamate release when administered 6 h after stress exposure. Thus, animals were subjected to FS, treated with ketamine (or saline) 6 h later, and sacrificed 24 h after stress exposure (Figure 1H). Acute ketamine brought back depolarization-evoked glutamate release in stressed animals to control levels (Figure 1J) [one-way ANOVA, *F*(2,20) = 8.40, *p* < 0.01; BPTH]. No effects were observed regarding basal glutamate release (Figure 1I) [one-way ANOVA, *F*(2,24) = 0.561, *p* > 0.05]. Again, basal and depolarization-evoked GABA releases were not affected (Supplementary Figures S1H–J).

Overall, these results show that acute ketamine injection, while inducing a transient increase of depolarization-evoked glutamate release (and possibly also basal glutamate release) in the PFC of naïve animals, blocks the raise of depolarization-evoked glutamate release induced by FS stress, both when administered at least 24 h before stress or 6 h after. To further investigate whether ketamine was able to rescue functional, morphological, and behavioral effects of acute stress, in subsequent experiments, we subjected the animals to FS and treated them with 10 mg/kg ketamine 6 h after. Because ketamine did not exert a major effect on glutamate release in naïve rats, except brief and transient increase (see above), in subsequent experiments, we did not use the group of control animals treated with ketamine.

### Subanesthetic Ketamine Restores the Increase of sEPSC Peak Amplitude in Prelimbic Prefrontal Cortex Layers II–III Pyramidal Neurons of Rats Subjected to Acute Footshock Stress

**FIGURE 2:**
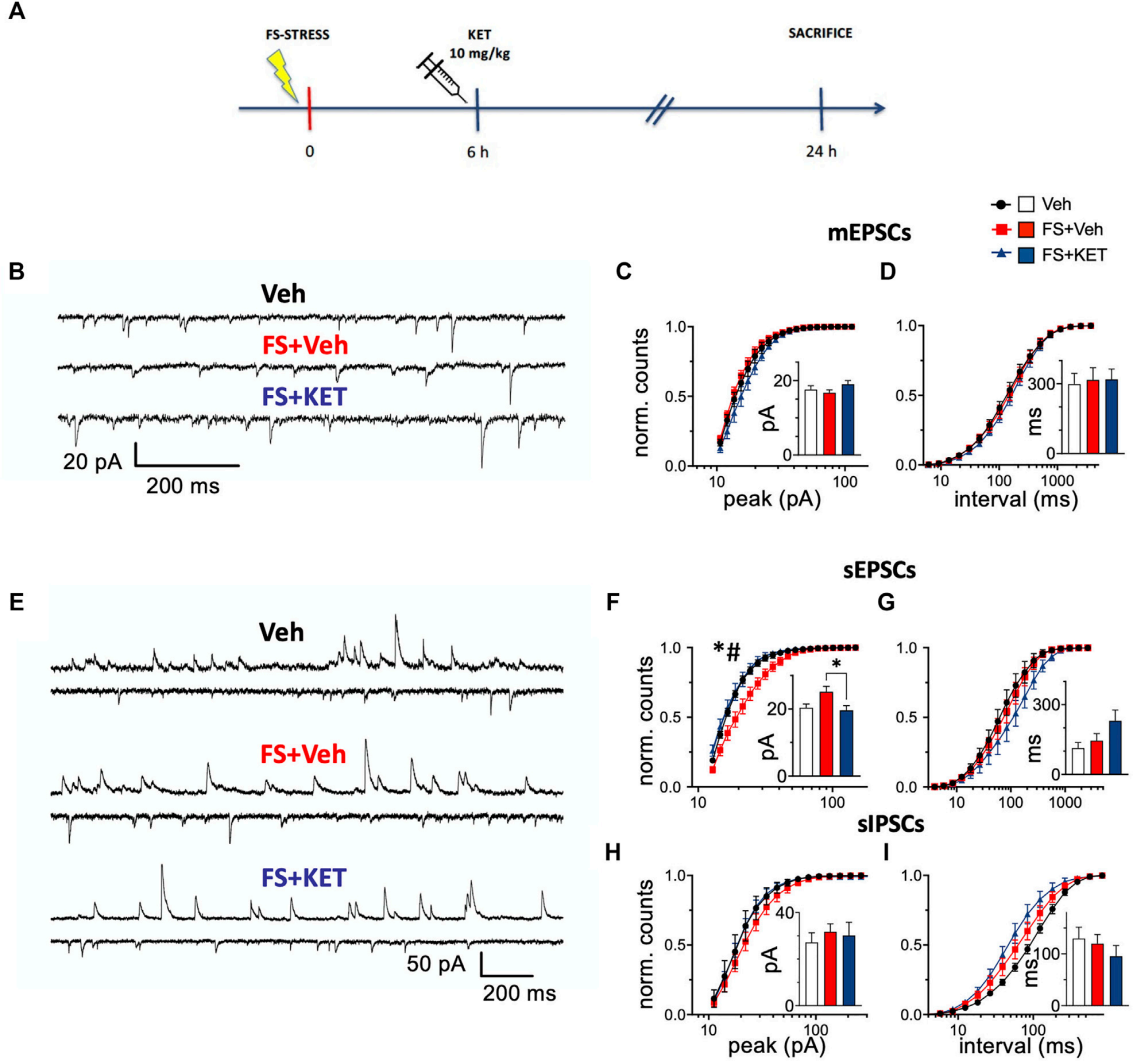
Miniature and spontaneous synaptic transmission. **(A)** Experimental plan timeline. Rats were injected with saline (Veh), subjected to acute FS, and injected with saline 6 h after stress (FS + Veh) or subjected to acute FS and injected with ketamine 6 h after stress (FS + KET). All the animals were sacrificed 24 h after FS beginning. **(B–D)** Miniature excitatory PSCs [mEPSCs, tetrodotoxin (TTX) 1 μM, picrotoxin 0.1 mM, and strychnine 1 μM] recorded with patch-clamp in putative pyramidal neurons within PL-PFC layers II–III. **(B)** Representative traces from Veh (upper trace), FS + Veh (middle trace), and FS + KET (bottom trace) cells (−60 mV). Normalized binned cumulative distributions of mEPSC peak amplitudes **(C)** and inter-event intervals **(D)** averaged across Veh (14 cells), FS + Veh (11 cells), and FS + KET (9 cells). Bar graph insets: means of the average mEPSC peak amplitude **(C)** and inter-event interval **(D)**. **(E–I)** Spontaneous excitatory and inhibitory PSCs (sEPSCs and sIPSCs respectively) PSCs. **(E)** Representative paired traces from Veh (top pair), FS + Veh (middle pair), and FS + KET (bottom pair) cells. In each pair, the upper and lower traces show sIPSCs (+3 mV) and sEPSCs (−58 mV), respectively. Normalized binned cumulative distributions of peak amplitudes **(F,H)** and inter-event intervals **(G,I)** of sEPSCs **(F,G)** and sIPSCs **(H,I)** averaged in Veh (sEPSCs: six cells; sIPSCs: five cells), FS + Veh (sEPSCs: six cells; sIPSCs: nine cells), and FS + KET (sEPSCs: seven cells; sIPSCs: eight cells). Bar graph insets: means of the average peak amplitude **(F,H)** and inter-event interval **(G,I)** of sEPSCs **(F,G)** and sIPSCs **(H,I)**. All the data are expressed as means ± SEM (see main text for more details). *Post-hoc* tests: *p < 0.05, FS + Veh vs Veh; #*p* < 0.05, FS + KET vs FS + Veh.

Having observed that acute ketamine was able to restore depolarization-evoked glutamate release to control levels in the PFC of FS-stressed rats, we next sought to investigate related alterations of synaptic transmission. To this end, we analyzed mEPSCs, sEPSCs, and sIPSCs in voltage-clamped pyramidal neurons within PL-PFC layers II–III of rats subjected to FS stress, treated with ketamine (or saline) 6 h later, and sacrificed 24 h after stress exposure (Figure 2).

mEPSCs (Figure 2B), generated by action potential-independent synaptic release, report about the number and properties of excitatory synaptic inputs (Kaeser and Regehr, 2014; Schneggenburger and Rosenmund, 2015; Kavalali, 2018; Williams and Smith, 2018). No main changes were found in mEPSC peak amplitude (Figure 2C). Two-way RM-ANOVA of binned peak distributions highlighted only a significant bin × group interaction [*F*(38, 589) = 1.55, *p* < 0.05], but with no significant *post-hoc* tests, and no significant effect of group [*F* (2,31) = 1.42], suggesting a slight trend towards increased peak amplitude in FS-stressed rats treated with ketamine (Figure 2C). Mean mEPSC peaks were not significantly different among the three experimental groups (bar graph inset in Figure 2C) [Veh = 17.6 ± 1.0 pA, FS + Veh = 16.7 ± 0.8 pA, FS + KET = 19.0 ± 1.0 pA, Kruskal–Wallis test (KWT) *p* > 0.05]. At the same time, no significant changes were found in mEPSC frequency (Figure 2D) [two-way RM-ANOVA of binned interval distributions: group *F*(2,31) = 0.251, bin × group *F*(38, 589) = 0.351; mean intervals: Veh = 298 ± 46 ms, FS + Veh = 316 ± 53 ms, FS + KET = 319 ± 45 ms, KWT *p* > 0.05).

sEPSCs (sIPSCs) (Figure 2E), recorded in the presence of a spontaneous spike activity in the surrounding neuronal network, provide a combined indication of properties of excitatory (inhibitory) synaptic inputs to the target neuron and of the firing levels in the local network (Béïque et al., 2004; Giusti et al., 2014). Two-way RM-ANOVA of binned distributions highlighted significant differences in sEPSC peak amplitude distributions (Figure 2F) [significant effect of group *F*(2,16) = 5.35, *p* < 0.05 and bin × group *F*(38, 304) = 4.79, *p* < 0.001], and *post-hoc* analysis showed that peak amplitude was higher in pyramidal neurons from FS-stressed rats compared to controls, and ketamine restored this change [Tukey’s multiple comparison test (TMPT)]. Accordingly, KWT confirmed the rescue of mean peak amplitude by acute ketamine in FS-stressed rats (bar graph inset in Figure 2F) (Veh = 20.4 ± 1.1 pA, FS + Veh = 25.1 ± 1.6 pA, FS + KET = 19.6 ± 1.4 pA, *p* < 0.05; Dunn’s multiple comparison test). No significant changes were instead found in sEPSC frequency (Figure 2G) [two-way RM-ANOVA of binned interval distributions: group *F*(2,16) = 1.14, bin × group *F*(38, 304) = 1.14; mean intervals: Veh = 113 ± 25 ms, FS + Veh = 145 ± 30 ms, FS + KET = 230 ± 46 ms, KWT *p* > 0.05].

sIPSC amplitude was not significantly different among groups (Figure 2H) [two-way RM-ANOVA of binned distributions: group *F*(2,19) = 0.708, bin × group *F*(38, 361) = 0.769; mean peaks: Veh = 27.2 ± 4.1 pA, FS + Veh = 31.7 ± 3.4 pA, FS + KET = 30.2 ± 5.7 pA, KWT *p* > 0.05], while a significant effect of bin × group interaction was found in sIPSC frequency, although with no significant *post-hoc* tests (Figure 2I) [two-way RM-ANOVA of binned interval distributions: bin × group *F*(38, 361) = 2.67, *p* < 0.001, no significant effect of group *F*(2,19) = 2.53]. This suggests a trend in increasing sIPSC frequency induced by both acute stress and ketamine administration. sIPSC mean intervals were not significantly different (bar graph inset in Figure 2I) (Veh = 130 ± 22 ms, FS + Veh = 120 ± 18 ms, FS + KET = 96 ± 21 ms, KWT *p* > 0.05).

Overall, the increase in peak sEPSC amplitude in PFC pyramidal neurons from FS-stressed rats and the restorative action of ketamine are in line with the effects observed in the measurement of glutamate release from synaptosomes.

### Subanesthetic Ketamine Restores the Changes in Apical Dendrite Length of Prelimbic PFC Layers II–III Pyramidal Neurons and Primary Neuronal Cultures Induced by Acute Stress or Corticosterone

**FIGURE 3:**
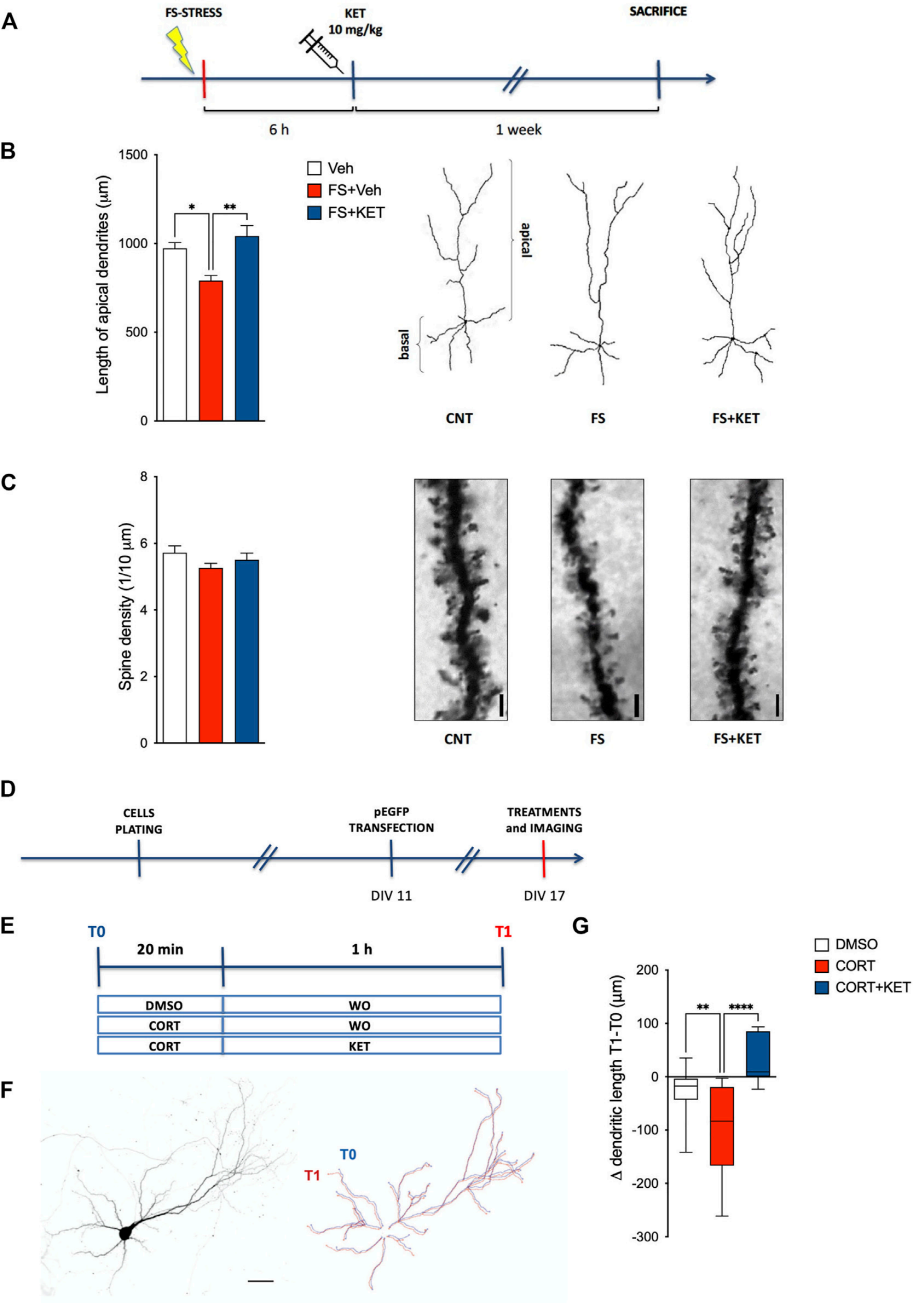
Dendritic arborization. **(A)** Experimental plan timeline for panels **(B,C)**. Rats were injected with saline (Veh), subjected to acute FS, and injected with saline 6 h after stress (FS + Veh) or subjected to acute FS and injected with ketamine 6 h after stress (FS + KET). All the animals were sacrificed 1 week after FS. Changes in the length of apical dendrites **(B)** and in the number of spines on apical dendrites **(C)** of pyramidal neurons within PL-PFC layers II–III. Data are expressed as means + SEM. *N* = 3–5 neurons/animal. One-way ANOVA, Bonferroni *post-hoc* test. *p < 0.05, **p < 0.01. Insets: representative drawings of PL-PFC layers II–III pyramidal neurons, as reconstructed with the “Simple Neurite Tracer” Fiji plugin **(B)** and representative light microscope images of dendritic segments analyzed; scale bar = 10 μm **(C)**. Timelines of the experimental plan **(D)** and of *in vitro* treatments **(E)** for panel **(G)**. Primary neuronal cultures were transfected with an enhanced green fluorescent protein (EGFP) expressing vector at day *in vitro* (DIV)11 and exposed to *in vitro* treatments at DIV17. For *in vitro* treatments, neurons were incubated with 500 nM corticosterone (CORT) or 0.2% DMSO for 20 min, followed by a wash-out (WO) with an astrocyte-conditioned medium or 1 µM ketamine (KET) for 1 h. Images were acquired before DMSO/CORT incubation (T0) and after WO/KET (T1). **(F)** Representative image of a cortical neuron in culture (left) and reconstruction of the dendritic tree at T0 (blue line) and T1 (red line) (right); scale bar = 50 μm. **(G)** Changes in the total dendritic length of EGFP-transfected pyramidal neurons in DIV17 primary neuronal cultures exposed to DMSO, corticosterone (CORT), or CORT and ketamine (CORT + KET). Data in the graph are expressed as box and whiskers (10–90 percentile) of the difference in dendritic length after treatments (T1) minus the length before treatments (T0). Ten–12 cells in two–three independent experiments. One-way ANOVA, Bonferroni *post-hoc* test. **p < 0.01, ****p < 0.0001.

In previous studies, we showed that acute FS stress causes a rapid (measured 24 h after stress) and long-lasting (up to at least 14 days) retraction of pyramidal neuron apical dendrites within layers II–III of PL-PFC (Nava et al., 2017). At the same time, we demonstrated that chronic pretreatment with a reference antidepressant (desipramine) prevented the stress-induced dendritic atrophy. Here, we investigated whether acute ketamine administration 6 h after FS exposure was able to prevent the retraction of dendritic arbor in PL-PFC layers II–III, as measured in animals sacrificed 7 days after the stress session (Figures 3A–C). Acute ketamine restored the reduction of the length of pyramidal neuron apical dendrites induced by FS (Figure 3B) [one-way ANOVA *F*(2,14) = 10.61, *p* < 0.01; BPHT), while no significant effect on dendritic spines was registered (Figure 3C) [one-way ANOVA *F*(2,15) = 1.55, *p* > 0.05).

To model in a simplified system, the neuronal effects of corticosterone, the major stress hormone, and ketamine and to directly test their action on dendritic remodeling, the results from *ex vivo* experiments were further validated in an *in vitro* model based on primary neuronal cultures briefly incubated with corticosterone and treated for 1 h with ketamine (Figures 3D–G). To this end, cortical neurons were transfected at low efficiency with an EGFP vector at DIV11 and imaged at DIV17 (Figure 3D), thus allowing us to obtain a few neurons expressing EGFP in a fully developed neuronal network. Twenty minutes of incubation with corticosterone caused a significant dendritic retraction in pyramidal neurons 1 h later, while the treatment with ketamine prevented dendritic retraction; a non-significant trend towards increased dendritic length in neurons treated with corticosterone and ketamine, compared to control, was also found (Figure 3G) [one-way ANOVA *F*(2,68) = 11.08, *p* < 0.001; BPHT].

### Subanesthetic Ketamine Facilitates the Extinction of Fear Memory in Rats Subjected to Acute Footshock Stress

**FIGURE 4:**
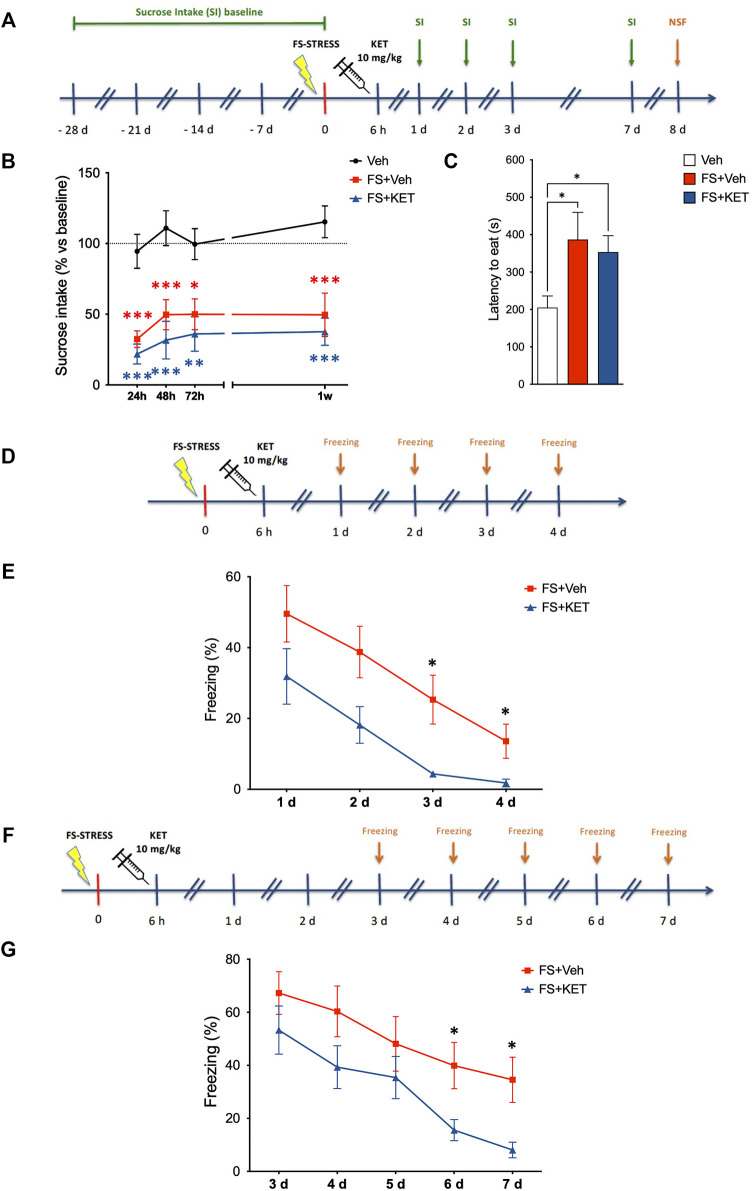
Behavioral studies. **(A)** Experimental plan timeline for panels **(B,C)**. Basal sucrose intake (SI) was established exposing the rats to SI test twice a week for 4 weeks. Animals were than subjected to FS and injected with saline (Veh) or ketamine (KET) 6 h after the beginning of the stress session. SI test was performed again 1, 2, 3, and 7 days after FS. The same rats were also exposed to the novelty-suppressed feeding (NSF) test 8 days after FS. Sucrose intake **(B)** and novelty-suppressed feeding **(C)** of rats injected with saline (Veh), subjected to acute FS, and injected with saline 6 h after stress (FS + Veh) or subjected to acute FS and injected with ketamine 6 h after stress (FS + KET). In **(B)**, changes in sucrose intake were measured 24 h, 48 h, 72 h and 1 week after stress exposure, and data are expressed as means + SEM of percent variation compared to baseline for each animal (see *Materials and Methods* for more details). Mixed-effects model, Tukey’s multiple comparison test. *p < 0.05, **p < 0.01, and ***p < 0.001 vs Veh. In **(C)**, data are expressed as mean latency to eat time(s) + SEM. One-way ANOVA, Bonferroni *post-hoc* test. *p < 0.05. **(D)** Experimental plan timeline for panel **(E)**. Rats were subjected to acute FS, injected with saline 6 h after stress (FS + Veh) or subjected to acute FS and injected with ketamine 6 h after stress (FS + KET), and placed in the FS cage for 5 min 1, 2, 3, and 4 days after the stress session (fear extinction sessions). The freezing time was measured. **(E)** Percent freezing time in the fear extinction sessions. Data are expressed as means + SEM. RM-ANOVA, Tukey’s multiple comparison test. *p < 0.05 vs FS + Veh. **(F)** Experimental plan timeline for panel **(G)**. Rats were subjected to acute FS, injected with saline 6 h after stress (FS + Veh) or subjected to acute FS and injected with ketamine 6 h after stress (FS + KET), and placed in the FS cage for 5 min 3, 4, 5, 6, and 7 days after the stress session (fear extinction sessions). The freezing time was measured. **(G)** Percent freezing time in the fear extinction sessions. Data are expressed as means + SEM. RM-ANOVA, Tukey’s multiple comparison test. *p < 0.05 vs FS + Veh.

Since we found that acute ketamine was able to rescue both functional glutamatergic alterations and the retraction of pyramidal neuron apical dendrites induced by acute FS stress in PFC, we then investigated whether these effects were accompanied by behavioral changes at the level of depressive/anxiety-like phenotypes and fear extinction (Figure 4). Depressive-like behavior was assessed by applying the classical sucrose intake test for anhedonia (Figures 4A,B). After habituation, all the animals were exposed to 1% sucrose for 1 h twice a week for 4 weeks, in order to determine a stable sucrose intake baseline for each rat. Animals were then exposed to sucrose 24 h, 48 h, 72 h, and 1 week after the stress session, and percent sucrose intake (vs relative baseline) was calculated for each animal. A mixed-effects model showed a significant effect of stress [*F*(2,30) = 33.65, *p* < 0.001; TMPT] (Figure 4B). Accordingly, the stressed animals displayed a remarkable reduction of sucrose intake already 24 h after stress and up to 1 week later; ketamine did not exert any restorative effect on this behavior.

Anxiety-like phenotype was also evaluated in the same animals using the NSF test, which was performed 1 week after FS exposure, 4 h after the last sucrose intake test (Figures 4A,C). Latency to eat was significantly higher in both stressed animals treated with vehicle or ketamine compared to controls, showing that ketamine did not rescue anxiety induced by FS in the NSF test (Figure 4C) [one-way ANOVA, *F*(2,31) = 6.85, *p* < 0.01; BPHT).

In a separate experiment, extinction of contextual fear memory was assessed by measuring the freezing time of rats when placed back in the same cage in which they had been exposed to FS (without receiving any shock) for four consecutive days after stress (Figure 4D). We found that, although all the animals (including those treated with ketamine) displayed freezing behavior, ketamine-treated animals extinguished fear before than vehicle-treated ones, since they did not show freezing anymore on the third and fourth day (while stressed animals treated with vehicle still displayed freezing behavior) (Figure 4E) [two-way RM-ANOVA, significant effect of subject *F*(22,66) = 4.855, *p* < 0.001; treatment *F*(1,22) = 7.360, *p* < 0.05; and time *F*(2.005,44.12) = 24.35, *p* < 0.001; TMCT).

To exclude that the effect of ketamine on fear memory in FS-stressed rats was dependent on memory deficits (i.e., the inability to associate the environment with shock danger), we performed the same fear extinction experiment again, placing the animals in the FS cage starting from 3 days after stress (when we registered a full recovery of freezing time in rats treated with ketamine in the first experiment) and then for the following 4 days (Figure 4F). Again, ketamine-treated animals showed reduced freezing, suggesting that they remember the cage as well as vehicle-treated animals, but dissociate the environment from the stress event before than vehicle-treated rats (Figure 4G) [two-way RM-ANOVA, significant effect of subject *F*(21,84) = 16.21, *p* < 0.001; treatment *F*(1,21) = 4.370, *p* < 0.05; and time *F*(2.453,51.52) = 28.45, *p* < 0.001; TMCT]. As a control of both experiments, we also tested the behavior of unstressed animals treated with vehicle; as expected, no freezing behavior was observed in these animals (Supplementary Figure S3).

## Discussion

In the present work, we found that, while acute ketamine only transiently and slightly affected glutamate release in the PFC of naïve animals, on the other hand, ketamine prevented (when administered 24 or 72 h before FS) and rescued (when administered 6 h after FS) the typical rise of depolarization-evoked glutamate release induced by acute inescapable FS. Because it would be clinically relevant to administer ketamine in the immediate aftermath of traumatic stress, we performed the complete study with rats treated 6 h after FS. The administration of ketamine 6 h after FS also rescued the increase of sEPSC peak amplitude in PL-PFC layers II–III pyramidal neurons. These functional synaptic changes were accompanied by a rescue of the stress-induced dendritic retraction of pyramidal neurons in PL-PFC layers II–III and, on the behavioral level, by facilitation of contextual fear extinction.

### Acute Ketamine Exerts Different Effects on Depolarization-Evoked Release of Glutamate in Naïve vs FS-Stressed Animals

The rapid antidepressant effect of ketamine has been traditionally associated with a burst of glutamate release in the PFC (Kavalali and Monteggia, 2012; Lener et al., 2017; Duman et al., 2019; Kadriu et al., 2019). This effect has been explained with disinhibition of presynaptic release of glutamate in the hippocampus and PFC (through the reduced activation of GABAergic neurons), thus leading to the activation of postsynaptic AMPA receptors and neurotrophic pathways (Lener et al., 2017; Zanos and Gould, 2018; Duman et al., 2019; Gould et al., 2019; Ali et al., 2020). An early study reported that subanesthetic ketamine increases glutamate outflow in the PFC, as measured by *in vivo* microdialysis in conscious naïve rats (Moghaddam et al., 1997). Subsequent studies showed that ketamine enhances EPSC responses induced by serotonin and/or hypocretin/orexin in medial PFC layer V pyramidal cells of naïve rats (Li et al., 2010), as well as rescuing the reduction of EPSC frequency/amplitude caused by chronic unpredictable stress (Li et al., 2011). In a recent study, we found that a single ketamine administration rescued the decrease of basal glutamate release from hippocampal synaptosomes of rats vulnerable to chronic mild stress (Tornese et al., 2019). Moreover, human proton magnetic resonance spectroscopy (1H-MRS) studies reported that ketamine administration increased cortical glutamate and glutamine levels in the anterior cingulate cortex (Stone et al., 2012) and reduced GABA levels in both the anterior cingulate cortex and HPC (Stone et al., 2012; Silberbauer et al., 2020), while a 3-T magnetic resonance spectroscopy study revealed only slight changes in the posterior cingulate cortex (Bednarik et al., 2021). Furthermore, increased PFC glutamate release after acute ketamine was also demonstrated by carbon-13 (13C) magnetic resonance spectroscopy, in both healthy and depressed subjects (Milak et al., 2016; Abdallah et al., 2018). Differently, Stan et al. (2014), by using *in vivo* measurement with microelectrode array, found that systemic subanesthetic S-ketamine reduced glutamate release in the subiculum, and local application reduced release in the subiculum and PL-PFC. More recently, by using an *in vitro* and *in vivo* combined approach (Fast Analytic Sensing Technology, FAST), ketamine was reported to reduce presynaptic glutamate neurotransmission in the subiculum and PL-PFC, with mechanisms implying retrograde stimulation of presynaptic adenosine A1 receptors (Lazarevic et al., 2021).

In the present study, we found that acute subanesthetic ketamine administration exerted different effects on PFC glutamate release in naïve rats compared to animals subjected to acute FS. In naïve rats, in line with previous evidence, we observed a transient rise of depolarization-evoked (and basal) glutamate release. This was different from the effect of acute desipramine, which induced a more sustained increase of basal glutamate release (up to 6 h) and no changes in depolarization-evoked release. Although we previously reported that chronic traditional antidepressants (including fluoxetine, reboxetine, and desipramine) decrease glutamate release in the hippocampus of naïve rats (Bonanno et al., 2005), to the best of our knowledge, we show here for the first time the effect of acute traditional antidepressant administration on glutamate release.

### Acute Ketamine Administered to Stressed Animals Stabilizes Dysfunctional Release of Glutamate

When ketamine was given before the exposure to acute FS, the effect on glutamate release was completely different from naïve animals. Indeed, no significant changes of basal release were observed in stressed animals (no increase of release 2 h after the injection as in control rats), while acute treatment with ketamine, 24 or 72 h before FS, prevented the increase of depolarization-evoked glutamate release induced by stress, as previously demonstrated for chronic traditional antidepressants with different primary mechanisms (Musazzi et al., 2010, 2013; Tardito et al., 2010). Interestingly, ketamine prevented the rise of glutamate release induced by acute stress only if administered at least 24 h before. Moreover, here we found that ketamine not only prevented the increase of depolarization-evoked glutamate release when administered before stress but also blocked the prolonged hyperactivation of glutamatergic synapses if administered 6 h after stress.

In previous studies, we have demonstrated that sEPSC amplitude is increased in PL-PFC immediately after FS exposure (Musazzi et al., 2010), and here, in line with the sustained increase of glutamate release observed up to 24 h after stress (Musazzi et al., 2017b), we found that the amplitude of sEPSCs was also significantly increased 24 h after FS (with no detectable sIPSC changes). Similar to glutamate release, ketamine injection 6 h after stress brought back sEPSC amplitude to control levels, thus preventing a sustained activation of excitatory transmission. We have also shown here that mEPSCs were unchanged in PL-PFC 24 h after FS, in contrast with the enhancement of mEPSC amplitude reported in prepubertal rats subjected to acute restraint stress (Yuen et al., 2009, 2011). This discrepancy could be dependent on the different types of stress applied and/or the age of the animals (adult animals in our study). Given the lack of FS effects on mEPSCs, it can be speculated that the increase of sEPSC amplitude may be dependent on higher firing in the neuronal network connected to the recorded neuron (Béïque et al., 2004; Giusti et al., 2014) and/or to selective regulation of action potential-dependent glutamate transmission (Calakos et al., 2004; Acuna et al., 2015; Robinson et al., 2019).

Altogether, the data combined from the present study and our previous work with chronic mild stress (Tornese et al., 2019) suggest that ketamine, rather than just inducing a general activation of glutamatergic synapses in corticolimbic areas, exerts a modulatory homeostatic effect on glutamatergic transmission, stabilizing glutamate dysfunction induced by either acute or chronic stress. Indeed, when synapses are hypofunctional (as in the case of chronic stress), ketamine restores glutamate release to control levels (Tornese et al., 2019) while, when glutamate release is enhanced by acute stress, ketamine dampens glutamate efflux, thus stabilizing glutamatergic transmission. Interestingly, glutamatergic dysfunction leading to impairment of synaptic homeostasis has been implicated in the pathophysiology of mood disorders, whereas the stabilization of glutamate transmission was proposed to be necessary for antidepressant action (Rubio-Casillas and Fernández-Guasti, 2016; Musazzi et al., 2018; Workman et al., 2018; Kavalali and Monteggia, 2020).

Previous compelling evidence have highlighted that the rapid and long-lasting antidepressant effect of ketamine is associated to (and dependent on) dendritic remodeling of pyramidal neurons in the PFC and hippocampus, with the rescue of dendritic atrophy and spine loss induced by the exposure to chronic stress (Li et al., 2011; Moda-Sava et al., 2019; Tornese et al., 2019; Wu et al., 2021). We have previously reported that the acute FS protocol we applied here induces marked and long-term neuroarchitectural changes in the brain (Treccani et al., 2014; Bonini et al., 2016; Nava et al., 2017; Musazzi et al., 2018; Chen F et al., 2020). In particular, acute stress causes a reduction of pyramidal neuron apical dendrite length within PFC layers II–III, which is measurable as soon as 24 h after stress exposure and up to 2 weeks afterwards. This reduction is prevented by chronic pretreatment with desipramine for 2 weeks (Nava et al., 2017).

Accordingly, in the present study, we observed a significant retraction of layers II–III pyramidal neuron dendrites 1 week after exposure to FS, and similar results were also obtained *in vitro*, incubating primary cortical neurons with the stress hormone corticosterone. The latter demonstrated that brief local application of corticosterone to neuronal cultures induces fast dendritic remodeling. Interestingly, together with inducing a functional stabilization of glutamatergic synapses, the administration of acute ketamine rescued dendritic retraction both in stressed animals (when administered 6 h after stress) and in neuronal cultures incubated with corticosterone. The present results clearly show that ketamine not only restores morphological alterations induced by chronic stress, as previously reported (Treccani et al., 2014; Nava et al., 2017; Musazzi et al., 2018; Chen F et al., 2020), but also the rapid and long-lasting dendritic remodeling caused by acute stress.

### Acute Ketamine May Facilitate Fear Extinction by Reducing Hyperactivation of Prelimbic PFC Induced by Acute Stress

Antidepressant/anxiolytic effects of ketamine have been previously explored mainly in chronic stress models (Ramaker and Dulawa, 2017; Pham and Gardier, 2019; Rincón-Cortés and Grace, 2020). Furthermore, in a study, ketamine was found to rescue the depressive-like phenotype induced by acute FS, when administered 24 h after stress exposure (Lee et al., 2019). Recent evidence suggested that ketamine may exert a prophylactic action against the effects of stressors, perhaps by enhancing resilience. It has been shown that a single administration of subanesthetic ketamine in mice 1 week in advance is protective against depressive-like behavior elicited by the social defeat stress protocol, learned helplessness protocol, or chronic corticosterone (Brachman et al., 2016). Ketamine was also tested in a contextual fear conditioning protocol, in which the administration 1 week before, but not after the protocol, reduced freezing behavior, facilitating fear extinction. Accordingly, it was suggested that ketamine is effective when administered before, but not after, exposure to stressors (McGowan et al., 2017). On the other hand, consistent with our results, a number of studies reported a reduction of fear both when ketamine was administered before (McGowan et al., 2017, 2018; Chen B. K et al., 2020) or after stress (Duclot et al., 2016; Girgenti et al., 2017; Asim et al., 2020; Radford et al., 2020), although some inconsistencies were found (Choi et al., 2020). Overall, this suggests that ketamine can affect previously established fear memory, thus improving the recovery from traumatic experiences. In the present work, looking at the behavioral outcomes of ketamine administration in the immediate aftermath of stress, we observed that, although it did not rescue anhedonic and anxious phenotypes in our model, the drug facilitated contextual fear extinction. Furthermore, we also found that the effect of subanesthetic ketamine on fear memory is not dependent on memory impairment, as it was consistently reported with abuse dissociative doses (Morgan et al., 2014). Interestingly, FS-stressed rats show long-lasting hyperactivation of glutamate release in PFC and an increase of sEPSC peak amplitude in PL-PFC 24 h after stress exposure, both rescued by ketamine. However, a limitation of this study is represented by the lack of the ketamine-only group; although the effect of ketamine on fear memory behavior is likely related to changes in glutamate release/transmission, in principle, other mechanisms cannot be excluded.

It has been shown that the rodent PL-PFC increases fear expression and inhibits extinction, while infralimbic (IL) PFC inhibits fear expression and increases extinction (Milad and Quirk, 2012). Therefore, the present results suggest that ketamine may facilitate fear extinction by reducing hyperactivation of PL-PFC induced by acute stress. Future work is warranted to assess whether ketamine also works by stimulating IL-PFC, as shown recently with optogenetics (Fuchikami et al., 2015).

## Conclusion

PTSD is a debilitating, often chronic and comorbid, mental illness that is difficult to treat and needs novel pharmacological strategies. In this context, recent evidence supports the potential benefit of ketamine (Krystal et al., 2018; Abdallah et al., 2019). Here, we found that subanesthetic ketamine administered before or after acute stress blocked the sustained activation of excitatory synapses in PFC, rescued the shrinkage of pyramidal neuron dendritic arbor in layer II/III, and favored the extinction of contextual fear memory in a rat model of PTSD. Although the molecular mechanisms involved in these effects require further investigation, our data concur to encourage the study of ketamine as an innovative therapeutic strategy to be administered shortly after the traumatic experience for inhibiting fear memory consolidation.

## Data Availability

The raw data supporting the conclusion of this article will be made available by the authors, without undue reservation.
