# Supplementary material for: Acute Ketamine Facilitates Fear Memory Extinction in a Rat Model of PTSD Along With Restoring Glutamatergic Alterations and Dendritic Atrophy in the Prefrontal Cortex
Source: Front Pharmacol. 2022 Mar 17;13:759626. doi: 10.3389/fphar.2022.759626 (PMC8968915; doi:10.3389/fphar.2022.759626)
Supplement: Supplementary file 1 [file DataSheet2.docx]

**SUPPLEMENTARY TABLES**

**Supplementary Table 1: N and statistics of Figure 1.**

| **Figure 1B** | | | | |
| --- | --- | --- | --- | --- |
| **Bonferroni's multiple comparisons test (Within each row, compare columns (simple effects within rows))** | **Significance** | **Adjusted**  **P Value** | **Experimental group** | **N** |
| **2 h** |  |  | **2 h** |  |
| Veh vs. DMI | * | 0.0182 | Veh | 14 |
| Veh vs. KET | ns | > 0.9999 | DMI | 9 |
| DMI vs. KET | * | 0.0421 | KET | 11 |
|  |  |  |  |  |
| **24 h** |  |  | **24 h** |  |
| Veh vs. DMI | ns | > 0.9999 | Veh | 14 |
| Veh vs. KET | ns | > 0.9999 | DMI | 7 |
| DMI vs. KET | ns | > 0.9999 | KET | 14 |
|  |  |  |  |  |
| **72 h** |  |  | **72 h** |  |
| Veh vs. DMI | ns | > 0.9999 | Veh | 14 |
| Veh vs. KET | ns | 0.0773 | DMI | 7 |
| DMI vs. KET | ns | 0.0931 | KET | 14 |

| **Figure 1C** | | | | |
| --- | --- | --- | --- | --- |
| **Bonferroni's multiple comparisons test (Within each row, compare columns (simple effects within rows))** | **Significance** | **Adjusted P Value** | **Experimental group** | **N** |
| **2 h** |  |  | **2 h** |  |
| Veh vs. DMI | ns | > 0.9999 | Veh | 12 |
| Veh vs. KET | * | 0.0481 | DMI | 10 |
| DMI vs. KET | ns | 0.5910 | KET | 17 |
|  |  |  |  |  |
| **24 h** |  |  | **24 h** |  |
| Veh vs. DMI | ns | > 0.9999 | Veh | 14 |
| Veh vs. KET | ns | > 0.9999 | DMI | 5 |
| DMI vs. KET | ns | > 0.9999 | KET | 15 |
|  |  |  |  |  |
| **72 h** |  |  | **72 h** |  |
| Veh vs. DMI | ns | > 0.9999 | Veh | 16 |
| Veh vs. KET | ns | 0.7791 | DMI | 7 |
| DMI vs. KET | ns | 0.8924 | KET | 15 |

| **Figure 1E** | | | | |
| --- | --- | --- | --- | --- |
| **Bonferroni's multiple comparisons test (Compare cell means regardless of rows and columns)** | **Significance** | **Adjusted P Value** | **Experimental group** | **N** |
| Veh vs. Veh+FS | *** | 0.0007 | Veh | 14 |
| Veh vs. KET | * | 0.0336 | Veh+FS | 6 |
| Veh vs. KET+FS | *** | 0.0002 | KET | 17 |
| Veh+FS vs. KET | * | 0.0493 | KET+FS | 7 |
| Veh+FS vs. KET+FS | ns | > 0.9999 |  |  |
| KET vs. KET+FS | * | 0.0175 |  |  |

| **Figure 1F** | | | | |
| --- | --- | --- | --- | --- |
| **Bonferroni's multiple comparisons test (Compare cell means regardless of rows and columns)** | **Significance** | **Adjusted P Value** | **Experimental group** | **N** |
| Veh vs. Veh+FS | * | 0.0106 | Veh | 14 |
| Veh vs. KET | ns | 0.4704 | Veh+FS | 5 |
| Veh vs. KET+FS | ns | 0.6134 | KET | 15 |
| Veh+FS vs. KET | * | 0.035 | KET+FS | 8 |
| Veh+FS vs. KET+FS | * | 0.0464 |  |  |
| KET vs. KET+FS | ns | 0.9184 |  |  |

| **Figure 1G** | | | | |
| --- | --- | --- | --- | --- |
| **Bonferroni's multiple comparisons test (Compare cell means regardless of rows and columns)** | **Significance** | **Adjusted P Value** | **Experimental group** | **N** |
| Veh vs. Veh+FS | *** | 0.0014 | Veh | 16 |
| Veh vs. KET | ns | > 0.9999 | Veh+FS | 6 |
| Veh vs. KET+FS | ns | > 0.9999 | KET | 15 |
| Veh+FS vs. KET | ** | 0.0096 | KET+FS | 6 |
| Veh+FS vs. KET+FS | * | 0.0486 |  |  |
| KET vs. KET+FS | ns | > 0.9999 |  |  |

| **Figure 1I** | | | | |
| --- | --- | --- | --- | --- |
| **Bonferroni's multiple comparisons test** | **Significance** | **Adjusted P Value** | **Experimental group** | **N** |
| Veh vs. FS+Veh | ns | 0.6909 | Veh | 9 |
| Veh vs. FS+KET | ns | 0.5234 | FS+Veh | 9 |
| FS+Veh vs. FS+KET | ns | 0.3042 | FS+KET | 9 |

| **Figure 1J** | | | | |
| --- | --- | --- | --- | --- |
| **Bonferroni's multiple comparisons test** | **Significance** | **Adjusted P Value** | **Experimental group** | **N** |
| Veh vs. FS+Veh | * | 0.0392 | Veh | 9 |
| Veh vs. FS+KET | ns | 0.1304 | FS+Veh | 8 |
| FS+Veh vs. FS+KET | ** | 0.0022 | FS+KET | 6 |

**Supplementary Table 2: N and statistics of Figure 2.**

| **Tukey’s multiple comparisons test (Within each row, compare columns (simple effects within rows))** | **Significance** | **Adjusted P Value** | **Experimental group** | **N** |
| --- | --- | --- | --- | --- |
|  |  |  |  |  |
| **Fig. 2C, all rows (1-20)** |  |  |  |  |
| Veh vs. FS+Veh | ns | ≥ 0.2183 | Veh | 14 |
| Veh vs. FS+KET | ns | ≥ 0.5152 | FS+Veh | 11 |
| FS+Veh vs. FS+KET | ns | ≥ 0.1390 | FS+KET | 9 |
|  |  |  |  |  |
| **Fig. 2D, all rows (1-20)** |  |  |  |  |
| Veh vs. FS+Veh | ns | ≥ 0.5934 | Veh | 14 |
| Veh vs. FS+KET | ns | ≥ 0.2124 | FS+Veh | 11 |
| FS+Veh vs. FS+KET | ns | ≥ 0.3499 | FS+KET | 9 |
|  |  |  |  |  |
| **Fig. 2F** |  |  |  |  |
| Veh vs. FS+Veh, rows 4 to 7 | * | ≤ 0.0452 | Veh | 6 |
| Veh vs. FS+KET, rows 1-20 | ns | ≥ 0.3026 | FS+Veh | 6 |
| FS+Veh vs. FS+KET, rows 1, 6-8 | * | ≤ 0.0403 | FS+KET | 7 |
|  |  |  |  |  |
| **Fig. 2G, all rows (1-20)** |  |  |  |  |
| Veh vs. FS+Veh | ns | ≥ 0.6124 | Veh | 6 |
| Veh vs. FS+KET | ns | ≥ 0.2089 | FS+Veh | 6 |
| FS+Veh vs. FS+KET | ns | ≥ 0.2499 | FS+KET | 7 |
|  |  |  |  |  |
| **Fig. 2H, all rows (1-20)** |  |  |  |  |
| Veh vs. FS+Veh | ns | ≥ 0.3717 | Veh | 5 |
| Veh vs. FS+KET | ns | ≥ 0.6152 | FS+Veh | 9 |
| FS+Veh vs. FS+KET | ns | ≥ 0.3188 | FS+KET | 8 |
| Fig. 2I, all rows (1-20) |  |  |  |  |
| Veh vs. FS+Veh | ns | ≥ 0.9985 | Veh | 5 |
| Veh vs. FS+KET | ns | ≥ 0.4013 | FS+Veh | 9 |
| FS+Veh vs. FS+KET | ns | > 0.9999 | FS+KET | 8 |

| **Dunn’s multiple comparisons test (Within each row, compare columns (simple effects within rows))** | **Significance** | **Adjusted P Value** | **Experimental group** | **N** |
| --- | --- | --- | --- | --- |
|  |  |  |  |  |
| **Fig. 2C, inset** |  |  |  |  |
| Veh vs. FS+Veh | ns | >0.9999 | Veh | 14 |
| Veh vs. FS+KET | ns | 0.3620 | FS+Veh | 11 |
| FS+Veh vs. FS+KET | ns | 0.1957 | FS+KET | 9 |
|  |  |  |  |  |
| **Fig. 2D, inset** |  |  |  |  |
| Veh vs. FS+Veh | ns | >0.9999 | Veh | 14 |
| Veh vs. FS+KET | ns | >0.9999 | FS+Veh | 11 |
| FS+Veh vs. FS+KET | ns | >0.9999 | FS+KET | 9 |
|  |  |  |  |  |
| **Fig. 2F, inset** |  |  |  |  |
| Veh vs. FS+Veh | ns | 0.1363 | Veh | 6 |
| Veh vs. FS+KET | ns | >0.9999 | FS+Veh | 6 |
| FS+Veh vs. FS+KET | * | 0.0318 | FS+KET | 7 |
|  |  |  |  |  |
| **Fig. 2G, inset** |  |  |  |  |
| Veh vs. FS+Veh | ns | >0.9999 | Veh | 6 |
| Veh vs. FS+KET | ns | 0.1519 | FS+Veh | 6 |
| FS+Veh vs. FS+KET | ns | 0.5131 | FS+KET | 7 |
|  |  |  |  |  |
| **Fig. 2H, inset** |  |  |  |  |
| Veh vs. FS+Veh | ns | >0.9999 | Veh | 5 |
| Veh vs. FS+KET | ns | >0.9999 | FS+Veh | 9 |
| FS+Veh vs. FS+KET | ns | 0.6834 | FS+KET | 8 |
|  |  |  |  |  |
| **Fig. 2I, inset** |  |  |  |  |
| Veh vs. FS+Veh | ns | >0.9999 | Veh | 5 |
| Veh vs. FS+KET | ns | 0.8042 | FS+Veh | 9 |
| FS+Veh vs. FS+KET | ns | >0.9999 | FS+KET | 8 |

**Supplementary Table 3: N and statistics of Figure 3.**

| **Figure 3B** | | | | |
| --- | --- | --- | --- | --- |
| **Bonferroni's multiple comparisons test** | **Significance** | **Adjusted P Value** | **Experimental group** | **N** |
| Veh vs. FS+Veh | * | 0.0129 | Veh | 6 |
| Veh vs. FS+KET | ns | 0.4646 | FS+Veh | 6 |
| FS+Veh vs. FS+KET | ** | 0.0017 | FS+KET | 5 |

| **Figure 3C** | | | | |
| --- | --- | --- | --- | --- |
| **Bonferroni's multiple comparisons test** | **Significance** | **Adjusted P Value** | **Experimental group** | **N** |
| Veh vs. FS+Veh | ns | 0.216 | Veh | 6 |
| Veh vs. FS+KET | ns | 0.6974 | FS+Veh | 6 |
| FS+Veh vs. FS+KET | ns | 0.6229 | FS+KET | 6 |

| **Figure 3G** | | | | |
| --- | --- | --- | --- | --- |
| **Bonferroni's multiple comparisons test** | **Significance** | **Adjusted P Value** | **Experimental group** | **N** |
| DMSO vs. CORT | ** | 0.0097 | DMSO | 22 |
| DMSO vs. CORT+KET | ns | 0.1089 | CORT | 23 |
| CORT vs. CORT+KET | **** | <0.0001 | CORT+KET | 11 |

**Supplementary Table 4: N and statistics of Figure 4.**

| **Figure 4B** | | | | |
| --- | --- | --- | --- | --- |
| **Tukey's multiple comparisons test Within each row, compare columns (simple effects within rows)** | **Significance** | **Adjusted**  **P Value** | **Experimental group** | **N** |
| **24 h** |  |  | **24 h** |  |
| Veh vs. FS+Veh | *** | 0.0001 | Veh | 13 |
| Veh vs. FS-KET | *** | <0.0001 | FS+Veh | 9 |
| FS+Veh vs. FS-KET | ns | 0.7712 | FS-KET | 10 |
|  |  |  |  |  |
| **48 h** |  |  | **48 h** |  |
| Veh vs. FS+Veh | ** | 0.0014 | Veh | 12 |
| Veh vs. FS-KET | *** | <0.0001 | FS+Veh | 6 |
| FS+Veh vs. FS-KET | ns | 0.7712 | FS-KET | 7 |
|  |  |  |  |  |
| **72 h** |  |  | **72 h** |  |
| Veh vs. FS+Veh | * | 0.0169 | Veh | 9 |
| Veh vs. FS-KET | ** | 0.0012 | FS+Veh | 5 |
| FS+Veh vs. FS-KET | ns | 0.7411 | FS-KET | 5 |
|  |  |  |  |  |
| **1 week** |  |  | **1 week** |  |
| Veh vs. FS+Veh | *** | 0.0008 | Veh | 9 |
| Veh vs. FS-KET | *** | <0.0001 | FS+Veh | 6 |
| FS+Veh vs. FS-KET | ns | 0.7842 | FS-KET | 10 |

| **Figure 4C** | | | | |
| --- | --- | --- | --- | --- |
| **Bonferroni's multiple comparisons test** | **Significance** | **Adjusted**  **P Value** | **Experimental group** | **N** |
| Veh vs. FS+Veh | * | 0.0484 | Veh | 6 |
| Veh vs. FS-KET | * | 0.0498 | FS+Veh | 5 |
| FS+Veh vs. FS-KET | ns | >0.9999 | FS-KET | 9 |

| **Figure 4E** | | | | |
| --- | --- | --- | --- | --- |
| **Tukey's multiple comparisons test** | **Significance** | **Adjusted**  **P Value** | **Experimental group** | **N** |
| **1 d** |  |  | **1 d** |  |
| FS+Veh vs. FS-KET | ns | 0.5082 | FS+Veh | 12 |
|  |  |  | FS-KET | 12 |
|  |  |  |  |  |
| **2 d** |  |  | **2 d** |  |
| FS+Veh vs. FS-KET | ns | 0.1281 | FS+Veh | 12 |
|  |  |  | FS-KET | 12 |
|  |  |  |  |  |
| **3 d** |  |  | **3 d** |  |
| FS+Veh vs. FS-KET | * | 0.0137 | FS+Veh | 12 |
|  |  |  | FS-KET | 12 |
|  |  |  |  |  |
| **4 d** |  |  | **4 d** |  |
| FS+Veh vs. FS-KET | * | 0.0456 | FS+Veh | 12 |
|  |  |  | FS-KET | 12 |

| **Figure 4G** | | | | |
| --- | --- | --- | --- | --- |
| **Tukey's multiple comparisons test** | **Significance** | **Adjusted**  **P Value** | **Experimental group** | **N** |
| **3 d** |  |  | **3 d** |  |
| FS+Veh vs. FS-KET | ns | >0.9999 | FS+Veh | 13 |
|  |  |  | FS-KET | 10 |
|  |  |  |  |  |
| **4 d** |  |  | **4 d** |  |
| FS+Veh vs. FS-KET | ns | 0.4105 | FS+Veh | 13 |
|  |  |  | FS-KET | 10 |
|  |  |  |  |  |
| **5 d** |  |  | **5 d** |  |
| FS+Veh vs. FS-KET | ns | >0.9999 | FS+Veh | 13 |
|  |  |  | FS-KET | 10 |
|  |  |  |  |  |
| **6 d** |  |  | **6 d** |  |
| FS+Veh vs. FS-KET | * | 0.0481 | FS+Veh | 13 |
|  |  |  | FS-KET | 10 |
|  |  |  |  |  |
| **7 d** |  |  | **7 d** |  |
| FS+Veh vs. FS-KET | * | 0.0383 | FS+Veh | 13 |
|  |  |  | FS-KET | 10 |

**Supplementary Table 5: N and statistics of Supplementary Figure 1.**

| **Supplementary Figure 1B** | | | | |
| --- | --- | --- | --- | --- |
| **Bonferroni's multiple comparisons test (Within each row, compare columns (simple effects within rows))** | **Significance** | **Adjusted P Value** | **Experimental group** | **N** |
| **2 h** |  |  | **2 h** |  |
| Veh vs. DMI | ns | >0.9999 | Veh | 7 |
| Veh vs. KET | ns | >0.9999 | DMI | 3 |
| DMI vs. KET | ns | >0.9999 | KET | 5 |
|  |  |  |  |  |
| **24 h** |  |  | **24 h** |  |
| Veh vs. DMI | ns | >0.9999 | Veh | 15 |
| Veh vs. KET | ns | >0.9999 | DMI | 7 |
| DMI vs. KET | ns | >0.9999 | KET | 14 |
|  |  |  |  |  |
| **72 h** |  |  | **72 h** |  |
| Veh vs. DMI | ns | >0.9999 | Veh | 11 |
| Veh vs. KET | ns | >0.9999 | DMI | 5 |
| DMI vs. KET | ns | >0.9999 | KET | 12 |

| **Supplementary Figure 1C** | | | | |
| --- | --- | --- | --- | --- |
| **Bonferroni's multiple comparisons test (Within each row, compare columns (simple effects within rows))** | **Significance** | **Adjusted P Value** | **Experimental group** | **N** |
| **2 h** |  |  | **2 h** |  |
| Veh vs. DMI | ns | >0.9999 | Veh | 17 |
| Veh vs. KET | ns | 0.9042 | DMI | 10 |
| DMI vs. KET | ns | >0.9999 | KET | 17 |
|  |  |  |  |  |
| **24 h** |  |  | **24 h** |  |
| Veh vs. DMI | ns | >0.9999 | Veh | 14 |
| Veh vs. KET | ns | >0.9999 | DMI | 7 |
| DMI vs. KET | ns | >0.9999 | KET | 14 |
|  |  |  |  |  |
| **72 h** |  |  | **72 h** |  |
| Veh vs. DMI | ns | >0.9999 | Veh | 13 |
| Veh vs. KET | ns | >0.9999 | DMI | 4 |
| DMI vs. KET | ns | >0.9999 | KET | 14 |
| DMI vs. KET | ns | >0.9999 | KET | 3 |

| **Supplementary Figure 1E** | | | | |
| --- | --- | --- | --- | --- |
| **Bonferroni's multiple comparisons test (Compare cell means regardless of rows and columns)** | **Significance** | **Adjusted P Value** | **Experimental group** | **N** |
| Veh vs. Veh+FS | ns | >0.9999 | Veh | 17 |
| Veh vs. KET | ns | >0.9999 | Veh+FS | 7 |
| Veh vs. KET+FS | ns | >0.9999 | KET | 17 |
| Veh+FS vs. KET | ns | >0.9999 | KET+FS | 6 |
| Veh+FS vs. KET+FS | ns | >0.9999 |  |  |
| KET vs. KET+FS | ns | >0.9999 |  |  |

| **Supplementary Figure 1F** | | | | |
| --- | --- | --- | --- | --- |
| **Bonferroni's multiple comparisons test (Compare cell means regardless of rows and columns)** | **Significance** | **Adjusted P Value** | **Experimental group** | **N** |
| Veh vs. Veh+FS | ns | 0.2972 | Veh | 14 |
| Veh vs. KET | ns | 0.397 | Veh+FS | 7 |
| Veh vs. KET+FS | ns | 0.3188 | KET | 14 |
| Veh+FS vs. KET | ns | 0.7227 | KET+FS | 6 |
| Veh+FS vs. KET+FS | ns | 0.9949 |  |  |
| KET vs. KET+FS | ns | 0.7308 |  |  |

| **Supplementary Figure 1G** | | | | |
| --- | --- | --- | --- | --- |
| **Bonferroni's multiple comparisons test (Compare cell means regardless of rows and columns)** | **Significance** | **Adjusted P Value** | **Experimental group** | **N** |
| Veh vs. Veh+FS | ns | >0.9999 | Veh | 13 |
| Veh vs. KET | ns | >0.9999 | Veh+FS | 9 |
| Veh vs. KET+FS | ns | >0.9999 | KET | 14 |
| Veh+FS vs. KET | ns | >0.9999 | KET+FS | 9 |
| Veh+FS vs. KET+FS | ns | >0.9999 |  |  |
| KET vs. KET+FS | ns | >0.9999 |  |  |

| **Supplementary Figure 1I** | | | | |  |
| --- | --- | --- | --- | --- | --- |
| **Bonferroni's multiple comparisons test** | **Significance** | **Adjusted P Value** | **Experimental group** | **N** | |
| Veh vs. FS+Veh | ns | >0.9999 | Veh | 10 | |
| Veh vs. FS+KET | ns | >0.9999 | FS+Veh | 10 | |
| FS+Veh vs. FS+KET | ns | >0.9999 | FS+KET | 9 | |

| **Supplementary Figure 1J** | | | | |  |
| --- | --- | --- | --- | --- | --- |
| **Bonferroni's multiple comparisons test** | **Significance** | **Adjusted P Value** | **Experimental group** | **N** | |
| Veh vs. FS+Veh | ns | 0.1113 | Veh | 10 | |
| Veh vs. FS+KET | ns | 0.6890 | FS+Veh | 9 | |
| FS+Veh vs. FS+KET | ns | 0.2360 | FS+KET | 9 | |

**Supplementary Table 6: N and statistics of Supplementary Figure 2.**

| **Supplementary Figure 2B** | | | | |
| --- | --- | --- | --- | --- |
|  | **Significance** | **Adjusted P Value** | **Experimental group** | **N** |
| **Kruskal-Wallis test** | * | 0.0383 |  |  |
| **Dunn’s multiple comparisons** |  |  |  |  |
| Veh vs. DMI | ns | >0.9999 | Veh | 5 |
| Veh vs. KET | * | 0.0495 | DMI | 3 |
| Veh vs. KET | ns | 0.3467 | KET | 4 |

| **Supplementary Figure 2C** | | | | |
| --- | --- | --- | --- | --- |
|  | **Significance** | **Adjusted P Value** | **Experimental group** | **N** |
| **Kruskal-Wallis test** | * | 0.0428 |  |  |
| **Dunn’s multiple comparisons** |  |  |  |  |
| Veh vs. DMI | * | 0.0488 | Veh | 3 |
| Veh vs. KET | ns | >0.9999 | DMI | 3 |
| DMI vs. KET | * | 0.0397 | KET | 3 |

| **Supplementary Figure 2D** | | | | |
| --- | --- | --- | --- | --- |
|  | **Significance** | **Adjusted P Value** | **Experimental group** | **N** |
| **Kruskal-Wallis test** | ns | 0.2321 |  |  |
| **Dunn’s multiple comparisons** |  |  |  |  |
| Veh vs. DMI | ns | 0.3032 | Veh | 3 |
| Veh vs. KET | ns | >0.9999 | DMI | 3 |
| DMI vs. KET | ns | 0.4081 | KET | 3 |

| **Supplementary Figure 2E** | | | | |
| --- | --- | --- | --- | --- |
|  | **Significance** | **Adjusted P Value** | **Experimental group** | **N** |
| **Kruskal-Wallis test** | ns | 0.6024 |  |  |
| **Dunn’s multiple comparisons** |  |  |  |  |
| Veh vs. DMI | ns | >0.9999 | Veh | 5 |
| Veh vs. KET | ns | >0.9999 | DMI | 3 |
| DMI vs. KET | ns | >0.9999 | KET | 4 |

| **Supplementary Figure 2F** | | | | |
| --- | --- | --- | --- | --- |
|  | **Significance** | **Adjusted P Value** | **Experimental group** | **N** |
| **Kruskal-Wallis test** | ns | 0.9929 |  |  |
| **Dunn’s multiple comparisons** |  |  |  |  |
| Veh vs. DMI | ns | >0.9999 | Veh | 3 |
| Veh vs. KET | ns | >0.9999 | DMI | 3 |
| DMI vs. KET | ns | >0.9999 | KET | 3 |

| **Supplementary Figure 2G** | | | | |
| --- | --- | --- | --- | --- |
|  | **Significance** | **Adjusted P Value** | **Experimental group** | **N** |
| **Kruskal-Wallis test** | ns | 0.4393 |  |  |
| **Dunn’s multiple comparisons** |  |  |  |  |
| Veh DMI | ns | 0.5391 | Veh | 3 |
| Veh vs. KET | ns | >0.9999 | DMI | 3 |
| DMI vs. KET | ns | >0.9999 | KET | 3 |
