# Supplementary material for: Acute Ketamine Facilitates Fear Memory Extinction in a Rat Model of PTSD Along With Restoring Glutamatergic Alterations and Dendritic Atrophy in the Prefrontal Cortex
Source: Front Pharmacol. 2022 Mar 17;13:759626. doi: 10.3389/fphar.2022.759626 (PMC8968915; doi:10.3389/fphar.2022.759626)
Supplement: Supplementary file 2 [file DataSheet1.docx]

## Supplementary Figures


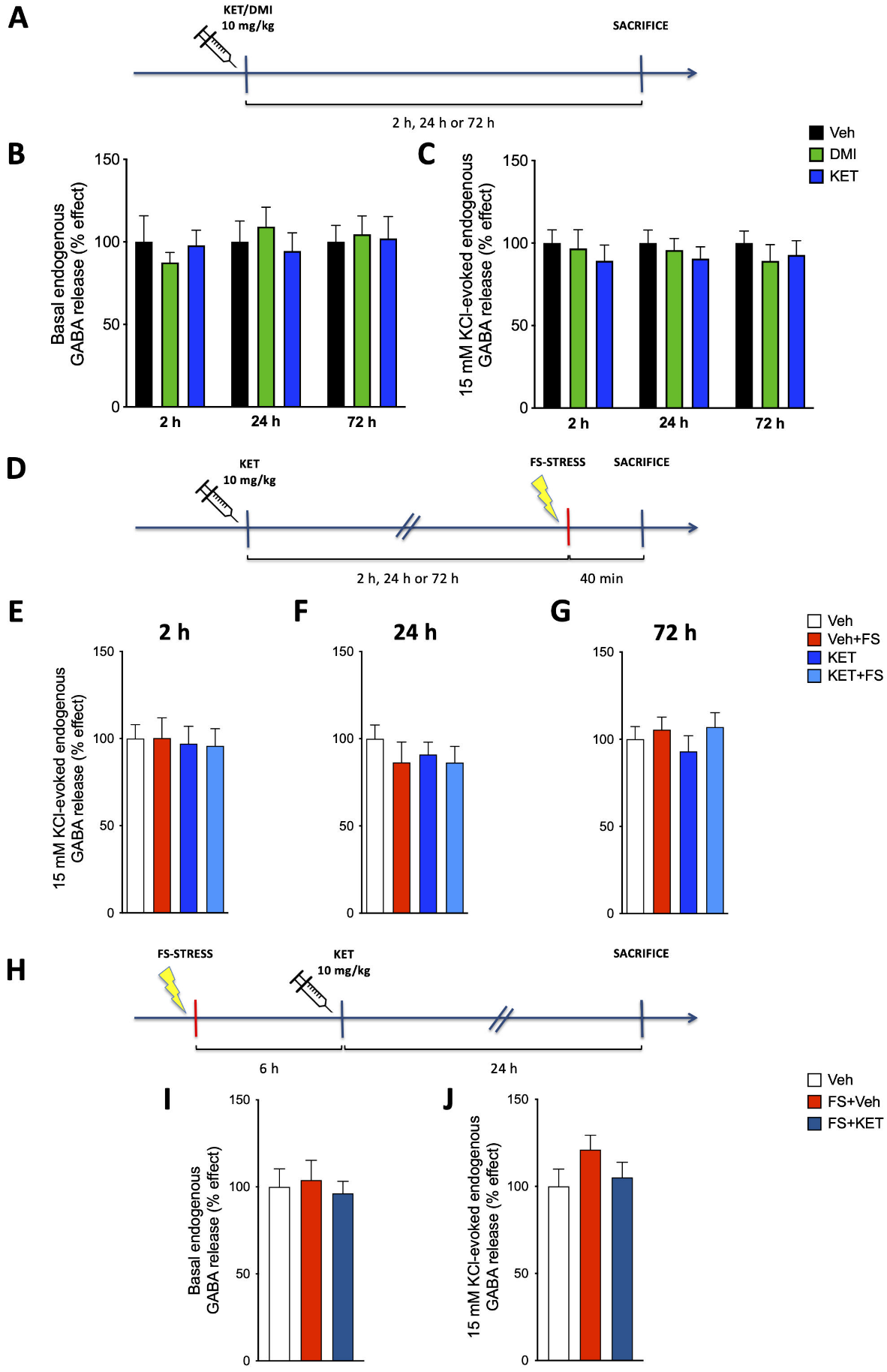


**Supplementary Figure 1 – Endogenous GABA release**

A: Experimental plan timeline for panels B and C. Rats were injected with saline (vehicle, Veh), ketamine (KET, 10 mg/kg) or desipramine (DMI, 10 mg/kg) and sacrificed 2, 24 or 72 h after.

Time-course of changes in basal (B) and 15 mM KCl-evoked (C) endogenous GABA release from PFC purified synaptosomes in superfusion. The net depolarization-evoked overflow was calculated by subtracting transmitter content of the basal outflow. Data are expressed as percent change means ± SEM vs. Veh. 2-way ANOVA, Bonferroni post-hoc test.

D: Experimental plan timeline for panels E-G. Rats were injected with saline (Veh), injected with saline and subjected to acute footshock (Veh+FS), injected with ketamine (KET), or injected with ketamine and subjected to acute FS (KET+FS). FS was administered 2, 24 or 72 h after Veh/KET injection and animals were sacrificed immediately after the FS session.

Changes in 15 mM KCl-evoked endogenous GABA release from PFC purified synaptosomes in superfusion of FS stressed rats measured 2 h (E), 24 h (F) or 72 h (G) after Veh/KET injection. Data are expressed as percent change means ± SEM vs. Veh. 1-way ANOVA, Bonferroni post-hoc test.

H: Experimental plan timeline for panels I and J. Rats were injected with saline (Veh), subjected to acute FS and injected with saline 6 h after stress (FS+Veh), or subjected to acute FS and injected with ketamine 6 h after stress (FS+KET). All the animals were sacrificed 24 h after FS beginning.

Changes in basal (I) and 15 mM KCl-evoked (J) endogenous GABA release from PFC purified synaptosomes in superfusion. Data are expressed as percent change means ± SEM vs. Veh. 1-way ANOVA, Bonferroni post-hoc test.


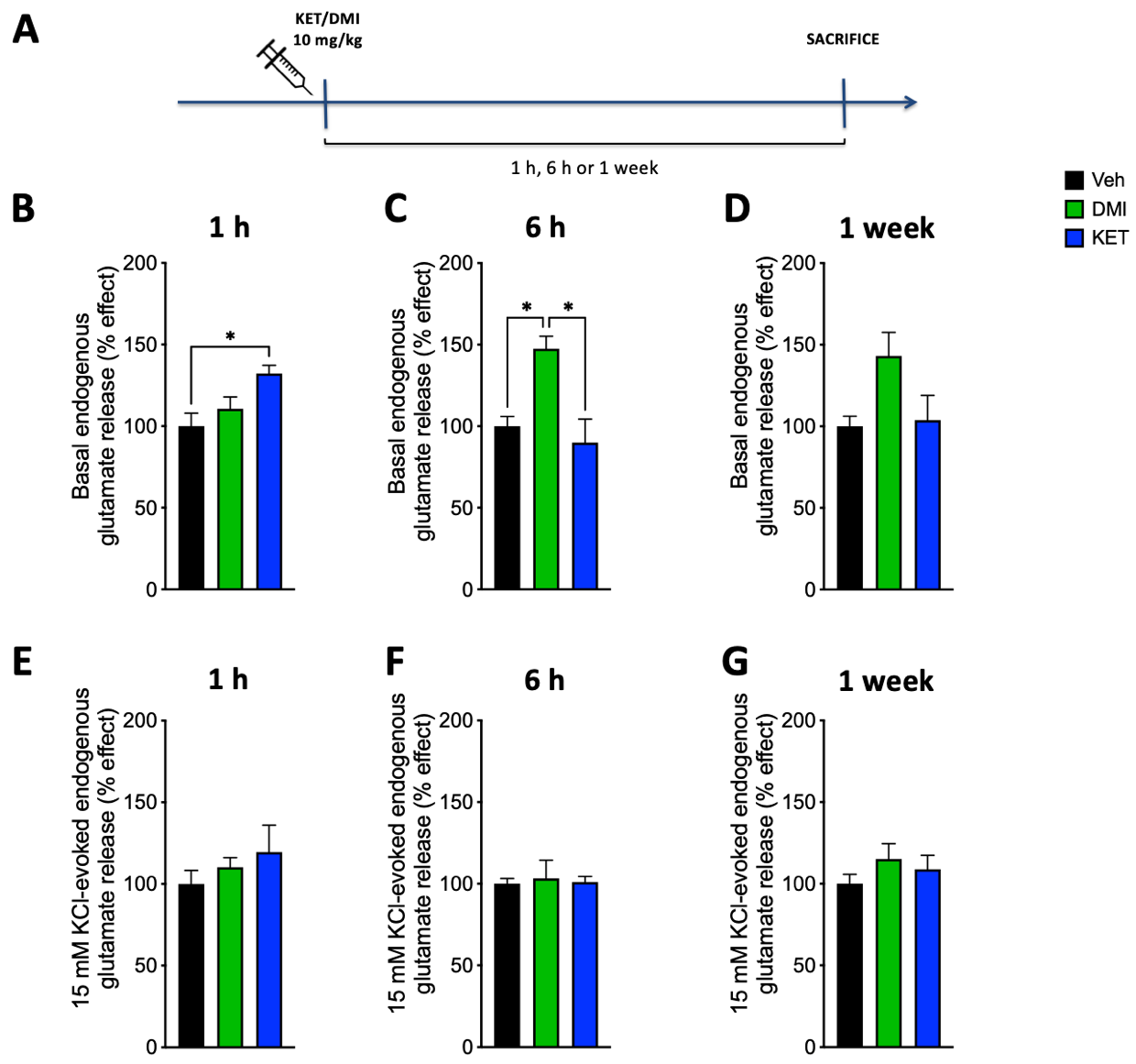


**Supplementary Figure 2 – Effect of ketamine and desipramine on endogenous glutamate release**

A: Experimental plan timeline. Rats were injected with saline (vehicle, Veh), ketamine (KET, 10 mg/kg) or desipramine (DMI, 10 mg/kg) and sacrificed 1 h, 6 h or 1 week after.

Time-course of changes in basal (B-D) and 15 mM KCl-evoked (E-G) endogenous glutamate release from PFC purified synaptosomes in superfusion of rats injected with saline (vehicle, Veh), ketamine (KET, 10 mg/kg) or desipramine (DMI, 10 mg/kg) and sacrificed 1 h (B, E), 6 h (C, F) or 1 week after (D, G). The net depolarization-evoked overflow was calculated by subtracting transmitter content of the basal outflow. Data are expressed as percent change means ± SEM vs. Veh. Kruskal-Wallis test, Dunn’s multiple comparison test. * p<0.05.


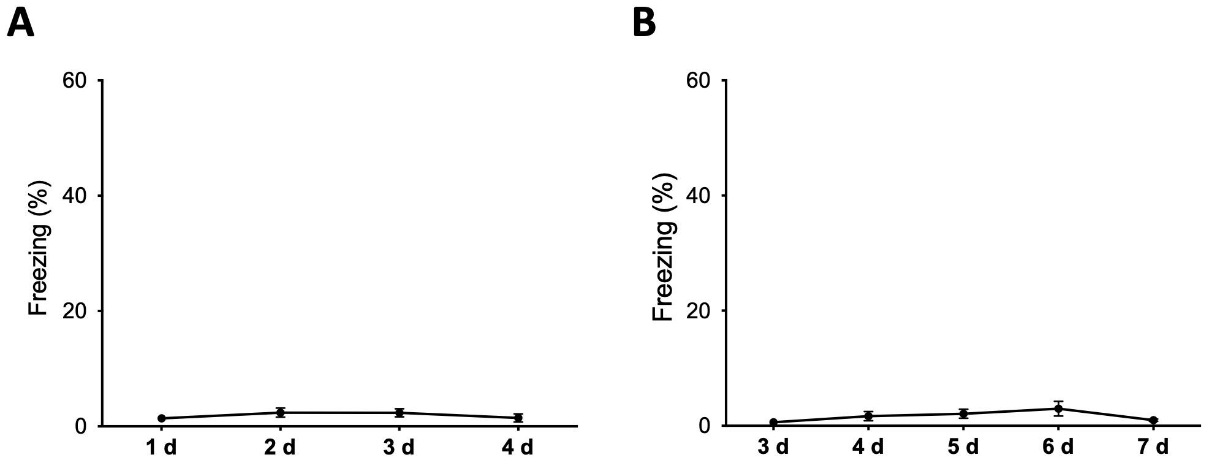


**Supplementary Figure 3 – Freezing behavior of unstressed rats.**

Rats were placed in the FS apparatus, without receiving the shock and placed in the same cage for 5 min 1, 2, 3 and 4 days after (A), or 3, 4, 5, 6 and 7 days after (B). Percent freezing time was measured. Data are expressed as means + SEM. RM-ANOVA, Tukey’s multiple comparison test.
